# Impact of spaceflight and artificial gravity on sulfur metabolism in mouse liver: sulfur metabolomic and transcriptomic analysis

**DOI:** 10.1038/s41598-021-01129-1

**Published:** 2021-11-08

**Authors:** Ryo Kurosawa, Ryota Sugimoto, Hiroe Imai, Kohei Atsuji, Koji Yamada, Yusuke Kawano, Iwao Ohtsu, Kengo Suzuki

**Affiliations:** 1 Euglena Co., Ltd., Minato-ku, Tokyo Japan; 2 Microalgae Production Control Technology Laboratory, RIKEN, Yokohama, Kanagawa Japan; 3 R&D Center for Tailor-Made-QOL, University of Tsukuba, Tsukuba, Ibaraki Japan; 4 Faculty of Life and Environmental Sciences, University of Tsukuba, Tsukuba, Ibaraki Japan; 5 Advanced Graduate Program for Future Medicine and Health Care, Tohoku University, Sendai, Miyagi Japan

**Keywords:** Metabolomics, Oxidoreductases, Transcriptomics, Astrobiology, Liver diseases

## Abstract

Spaceflight induces hepatic damage, partially owing to oxidative stress caused by the space environment such as microgravity and space radiation. We examined the roles of anti-oxidative sulfur-containing compounds on hepatic damage after spaceflight. We analyzed the livers of mice on board the International Space Station for 30 days. During spaceflight, half of the mice were exposed to artificial earth gravity (1 g) using centrifugation cages. Sulfur-metabolomics of the livers of mice after spaceflight revealed a decrease in sulfur antioxidants (ergothioneine, glutathione, cysteine, taurine, thiamine, etc.) and their intermediates (cysteine sulfonic acid, hercynine, *N*-acethylserine, serine, etc.) compared to the controls on the ground. Furthermore, RNA-sequencing showed upregulation of gene sets related to oxidative stress and sulfur metabolism, and downregulation of gene sets related to glutathione reducibility in the livers of mice after spaceflight, compared to controls on the ground. These changes were partially mitigated by exposure to 1 g centrifugation. For the first time, we observed a decrease in sulfur antioxidants based on a comprehensive analysis of the livers of mice after spaceflight. Our data suggest that a decrease in sulfur-containing compounds owing to both microgravity and other spaceflight environments (radiation and stressors) contributes to liver damage after spaceflight.

## Introduction

Spaceflight is known to impose changes on human physiology with unknown molecular etiologies because of hostile environments, such as microgravity and high-dose space radiation. Multi-omics and systems biology studies using data from astronauts and hundreds of samples flown into space revealed that the liver undergoes a larger change in the expression levels of genes and proteins than other organs^1^. Research on astronaut health and model organisms has revealed several features of molecular changes during space travel, including oxidative stress^2^.

Oxidative stress is caused by an altered balance between the production of reactive oxygen and/or nitrogen species (ROS, RNS) and the antioxidant defense capacity partly achieved by sulfur-containing compounds^3^. Exposure to space radiation, hypoxia, and microgravity induces the production of ROS and RNS^1^, and sulfur-containing compounds protect against the oxidative stress caused by radiation^4^. Furthermore, accumulating evidence indicates that alterations in the metabolism of sulfur-containing amino acids are important factors contributing to the development of liver disease^5^. Using transcriptomic analyses, changes in gene expression related to sulfur metabolism have been detected in the livers of mice exposed to space^1,6–8^.

Sulfur metabolism involves a variety of reactions, forming a complex metabolic network^3^. In mammals, the liver plays a central role in sulfur metabolism because nearly half of the dietary methionine, an essential sulfur-containing amino acid, is metabolized there^9^. Methionine metabolism occurs primarily via the trans-sulfuration pathway, which results in the transfer of methionine sulfur to serine to form cysteine^10^. l-Cysteine plays an important role in this network as a metabolic hub, and its reduced sulfur has strong nucleophilic properties^11–13^. An important role of sulfur in biological systems is that it controls the redox state in the cells; for example, glutathione (GSH), which is an abundant tripeptide (0.5–10 mmol/L) comprising glutamate, cysteine, and glycine, reduces reactive oxygen species as the most common defense mechanism against free radical damage^14^. Dysregulation of GSH synthesis contributes to the pathogenesis of liver diseases^15^. Moreover, ergothioneine, a ubiquitous sulfur-containing compound (100–2 mmol/L) in animals, is synthesized only by a few microorganisms and acts as an antioxidant and cytoprotectant^16,17^. Furthermore, taurine is an abundant amino sulfonic acid with diverse cytoprotective activities, including antioxidant activity^18^. Importantly, administration of GSH, ergothioneine and taurine has protected against liver damage in vivo^19^. In general metabolomes, it had been considered extremely difficult to measure highly reactive sulfur metabolites, such as cysteine and glutathione, and inorganic sulfur compounds, such as sulfite, hydrogen sulfide, and thiosulfate. Recently, the measurements of reactive sulfur compounds have been broken through by the multiple-reaction monitoring mode (MRM) of mass spectrometry using alkylating reagents, such as monobromobimane^20^. This method has been used to detect and compare approximately 61 chemical compounds related to sulfur metabolism in humans^21,22^.

Thus, in this study, we aimed to investigate the changes in sulfur compounds in the livers of mice after spaceflight and those of controls on the ground. We obtained the livers of mice under microgravity in space, mice under artificial earth gravity (1 g) in space, and the control mice on Earth, provided by the Japan Aerospace Exploration Agency (JAXA) mission at the International Space Station (ISS) in 2017 (Mouse habitat Unit-2; MHU-2)^23^. In the mission, JAXA used a newly established experimental platform known as the Multiple Artificial-gravity Research System (MARS)^24–29^, which monitors the effect of altered gravity on live mice by centrifuging mouse cages to control the gravity experienced. JAXA conducted space experiments using MARS in the ISS during orbit for 30 days, and then returned all the mice back to Earth in a live condition. The environmental conditions of controls on the ground were as close as possible to the acclimation conditions of mice used in flight, using the same diets and the same type of cages. Notably, exposure to 1 g during spaceflight is expected to help elucidate the influence of microgravity on mice. Indeed, artificial 1 g has mitigated some of the harmful effects of microgravity during spaceflight, such as a decrease in femur bone density and soleus/gastrocnemius muscle mass (MHU-1), downregulation of erythrocyte-related genes in the spleen (MHU-1), retinal endothelial cell damage (MHU-1), and reduction in thymic size (MHU-1 and MHU-2)^24–26,28^. Moreover, artificial 1 g tended to mitigate a decrease in body weight (MHU-2)^23^. Importantly, Oil Red O (ORO) staining of liver samples showed increased lipid droplets in mice after spaceflight compared to ground controls, though there was no artificial 1 g group in this session (MHU-3)^30^. Our results showed that spaceflight decreased sulfur metabolites, including ergothioneine, GSH, and cysteine, in the livers of mice. Furthermore, RNA sequencing demonstrated a change in the gene expression of multiple pathways related to sulfur-containing compounds. This study is the first to perform a comprehensive analysis of sulfur-containing compounds in the liver after spaceflight.

## Results

### Sulfur metabolomics of the livers from mice exposed to spaceflight

**Figure 1.**
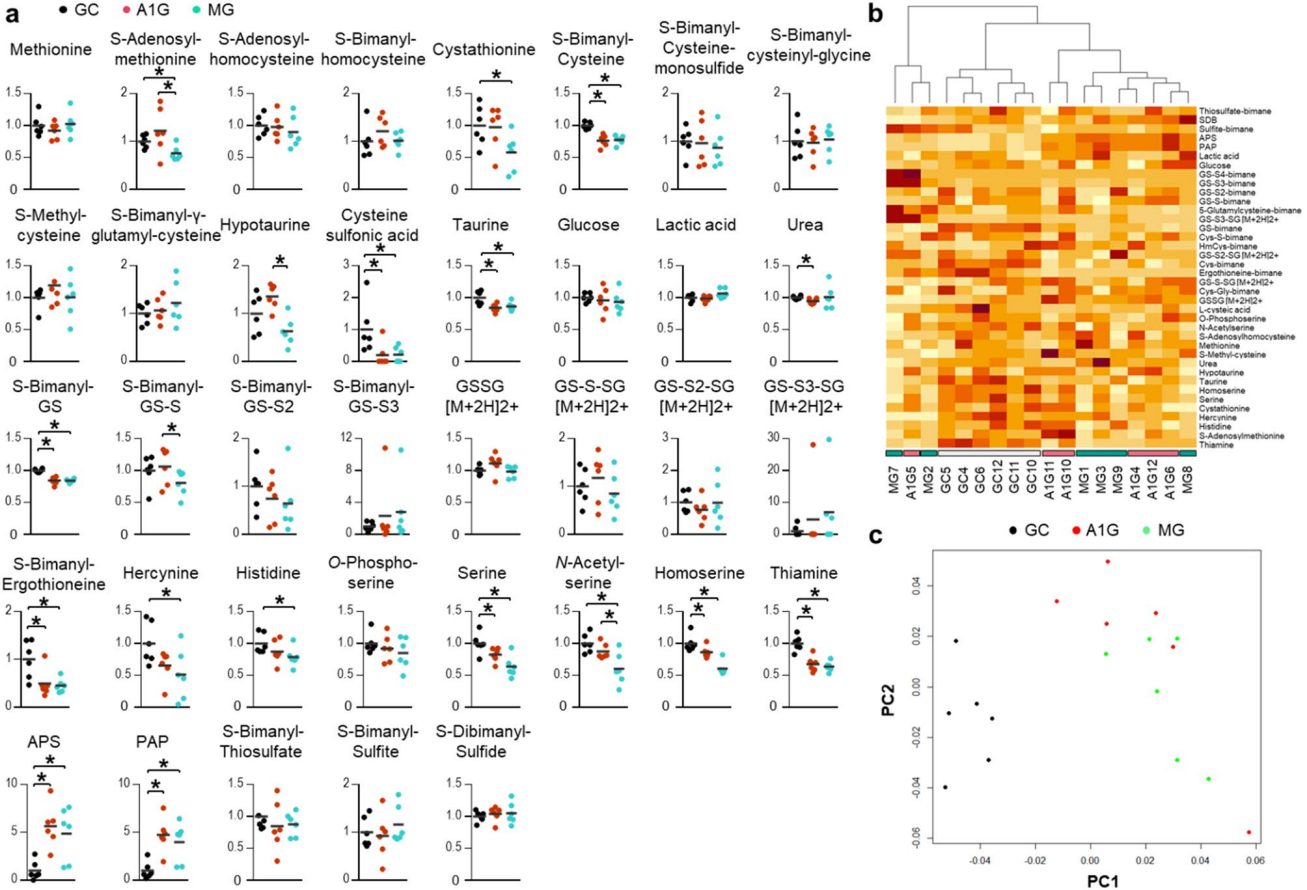
Measurement of sulfur compounds in the liver. (**a**) Quantification of sulfur compounds in the liver homogenates of control mice on Earth (GC), mice under artificial earth-gravity in space (A1G), and mice under microgravity in space (MG). GS, Glutathione; GSSG, glutathione disulfide; PAP, 3′-phosphoadenosine-5′-phosphosulfate; APS, adenosine-5′-phosphosulfate. ‘S-Bimanyl-’ indicates that the compounds were detected as bimane-derivates. Each horizontal bar represents the mean. *P < 0.05. Comparisons of parameters were performed with one-way ANOVA followed by Dunnett’s test for multiple comparisons (*n *= 6 each). Dot plots were made using GraphPad Prism 6.07 for Windows, GraphPad Software, San Diego, CA USA, www.graphpad.com. (**b**) Heatmap and hierarchical clustering were performed in R. The red color intensity correlates with the relative signal abundance. (**c**) Spatial placement of crowds on a non-metric multidimensional scaling (NMDS) using the Bray index. NMDS analysis, using the “vegan” package in R to analyze similarity indices, was performed on normalized metabolomics data that was subsequently log-transformed and auto-scaled^31,32^. The first two coordinates were plotted. *PC* principal coordinate.

To study the regulation of sulfur-containing compounds after spaceflight, we conducted sulfur metabolomics of the livers from the control mice on Earth (GC mice), mice under artificial earth gravity in space (A1G mice), and mice under microgravity in space (MG mice). Thirty-eight compounds were detected in the relative quantification of sulfur-containing compounds using sensitive liquid chromatography coupled to tandem mass spectrometry (LC–MS/MS), of which half were significantly different between GC and MG mice, and between GC and A1G mice (Fig. 1a). Spaceflight decreased S-adenosylmethionine, cystathionine, cysteine, hypotaurine, cysteine sulfonic acid, taurine, GSH, glutathione persulfide (GS-SH), ergothioneine, hercynine, histidine, serine, *N*-acetylserine, homoserine, and thiamine, whereas some sulfur compounds were decreased only in MG mice compared to GC or A1G mice. Conversely, spaceflight increased adenosine-5′-phosphosulfate (APS) and 3′-phosphoadenosine-5′-phosphosulfate (PAP). Heatmap analysis showed that the amount of sulfur-containing compounds in the livers of A1G and MG mice was considerably different from that of GC mice (Fig. 1b). Similarly, a multivariate sample-to-sample similarity mapping analysis based on the detected sulfur-related compound data showed that the sulfur metabolomics profiles of A1G and MG mice were considerably different from those of GC mice (Fig. 1c). The correlation coefficients of each compound calculated for the two coordinates in the non-metric multidimensional scaling showed that GSH, thiamine, and cysteine were the main contributors to PC1, and glutathione disulfide (GSSG), the oxidized form of GSH, was the main contributor to PC2 (Supplementary Table 1 online).

**Figure 2:**
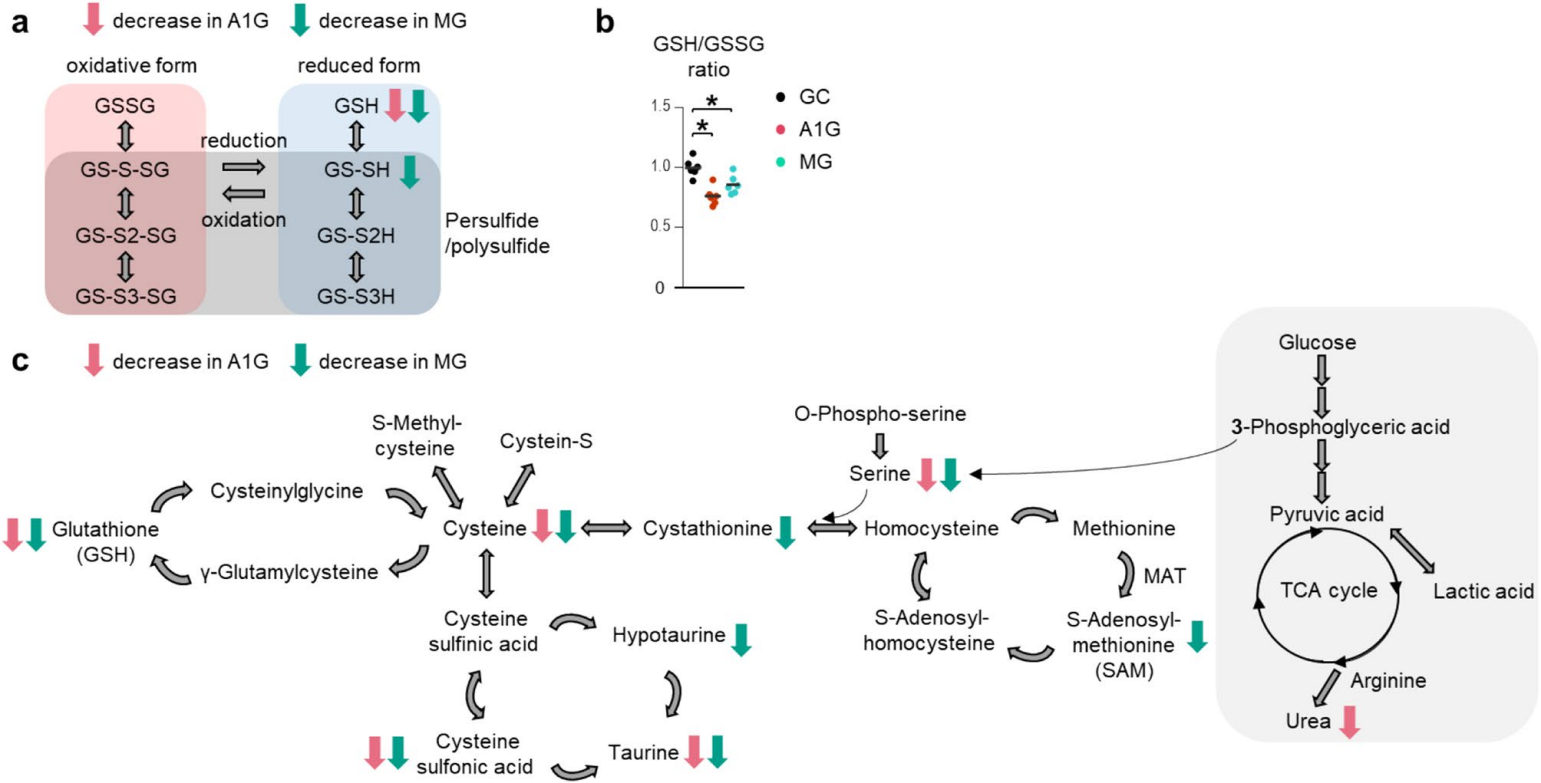
Downregulation of sulfur compounds in the livers of mice exposed to a spaceflight. (**a**) An illustration of the reduction and oxidization network of glutathione. Glutathione (GSH), glutathione disulfide (GSSG), and their persulfidated derivatives GS-SH, GS-S2H, GS-S3H, GS-S-SG, GS-S2-SG, and GS-S3-SG.  or  indicates a significant decrease in A1G or MG mice compared to GC mice. GC, control mice on Earth; A1G, mice under artificial earth gravity in space; MG, mice under microgravity in space. (**b**) The ratio of the relative amount of GSH to GSSG. Each horizontal bar represents the mean. Dot plots were made using GraphPad Prism 6.07 for Windows, GraphPad Software, San Diego, CA USA, www.graphpad.com. (**c**) An illustration of the metabolic network of sulfur compounds in mammals.  and  between compounds indicates metabolic reactions in mammals.  or  indicates a decrease in A1G or MG mice compared to GC mice. MAT, methionine adenosyltransferase. *P < 0.05. Comparisons of parameters were performed with one-way ANOVA followed by Dunnett’s test for multiple comparisons (*n *= 6 each).

GSH is converted to an oxidized form of GSH (GSSG), which is formed by a covalent disulfide bond between two GSHs and can be restored to separate GSHs by the activity of NADPH-glutathione reductase^3,14^. To evaluate the redox status of the liver after spaceflight, we focused on the increase and decrease in GSH-related compounds in the sulfur metabolomics data (Fig. 2a). GSH decreased after spaceflight, and GS-SH (persulfide form of GSH) decreased in MG mice, whereas GS-S2H (persulfide form of GS-SH) and GS-S3H (persulfide form of GS-S2H) did not change significantly. Here, GS-SH exhibits cytoprotective effects against oxidative stress^33^. In contrast, there was no significant change in GSSG, GS-S-SG (a persulfide form of GSSG), GS-S2-SG (a persulfide form of GS-S-SG), and GS-S3-SG (a persulfide form of GS-S2-SG). Importantly, the ratio of GSH/GSSG was lower in A1G and MG mice than in GC mice, indicating a shift to an oxidized form (Fig. 2b). Thus, our quantification of GSH-related compounds indicates that their reduced forms decreased after spaceflight with a shift to their oxidized forms. Similarly, hercynine is the oxidized form of ergothioneine^34^, and the ratio of hercynine to ergothioneine was higher (not significantly) in A1G and MG mice than in GC mice (Supplementary Fig. S1 online).

We then examined the metabolic network of sulfur compounds in mammals with reference to previous research^35^ (Fig. 2c). Our sulfur metabolomics data indicated that cysteine, GSH, serine, cysteine sulfonic acid, and taurine decreased in the livers of A1G and MG mice compared to those in GC mice. Furthermore, S-adenosylmethionine (SAM), cystathionine, and hypotaurine were downregulated only in MG mice compared to GC or A1G mice, indicating that the difference in gravity between A1G and MG mice affected the amount of these compounds. Notably, SAM is an important precursor of cysteine and GSH, especially in the liver^5^. Here, the fact that there was no decrease in methionine in A1G and MG mice compared to that in GC mice, which is not synthesized de novo in mammals and is a substrate for other sulfur-containing compounds, indicates that the dietary intake of methionine in space was sufficient. Furthermore, changes were observed in ergothioneine and other sulfur-containing compounds that are biosynthesized only by microorganisms and plants (Supplementary Fig. S2 online)^21,36^.

Altogether, multivariate analysis showed that the sulfur metabolomics profiles of A1G and MG mice were considerably different from those of GC mice, and that sulfur antioxidants such as ergothioneine, GSH, and cysteine were downregulated in the livers of A1G and MG mice compared to those in GC mice with a shift to an oxidized state, whereas some sulfur compounds were downregulated only in MG mice compared to GC or A1G mice.

### Transcriptomic analysis of the livers from mice exposed to spaceflight

**Figure 3.**
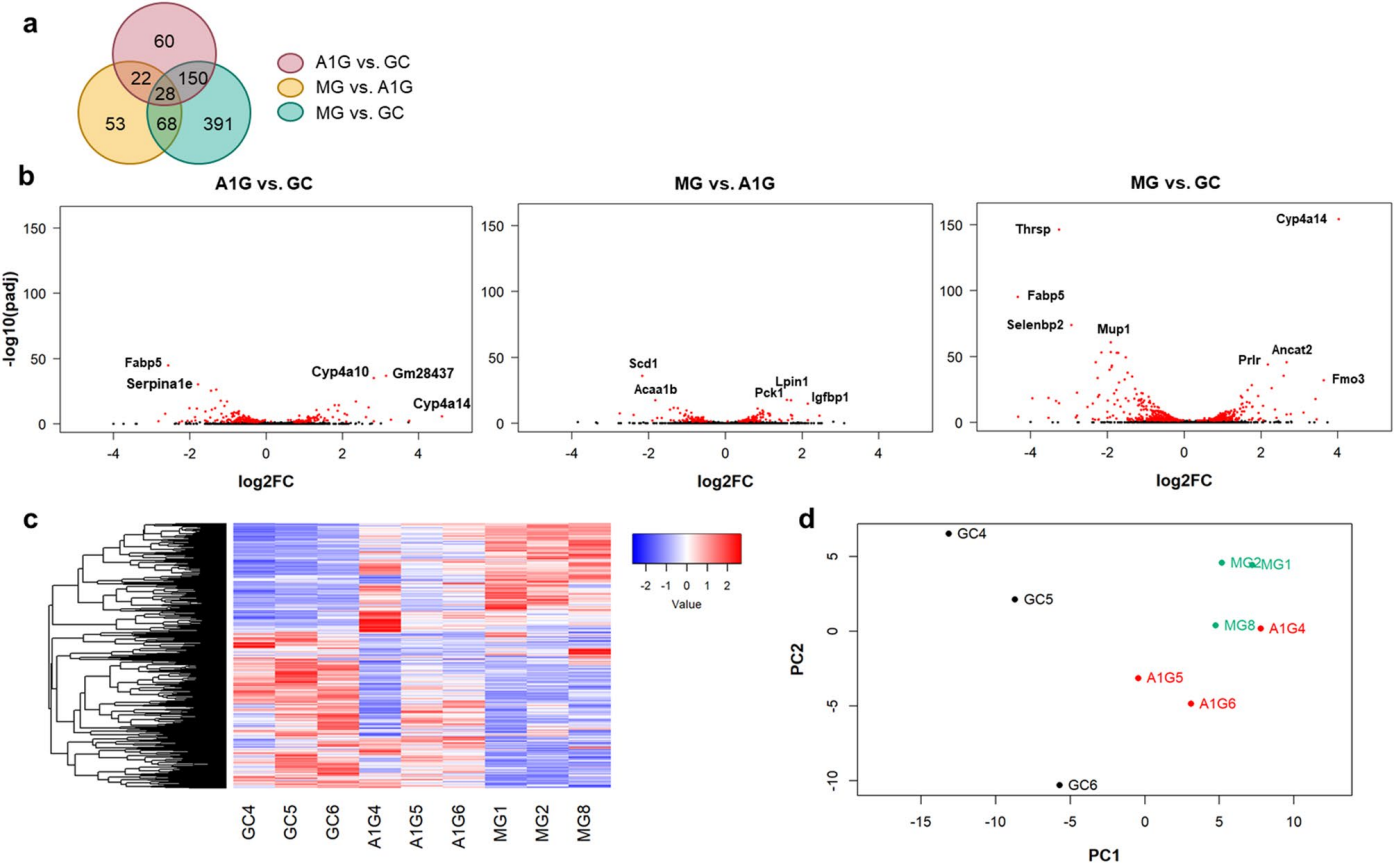
Global transcriptomic differences in the livers from control mice on Earth (GC), mice under artificial earth gravity in space (A1G), and mice under microgravity in space (MG). (**a**) Venn diagram representing the number of significant differentially expressed genes. The number of overlapping regions means significant differentially expressed genes in common. For example, the number of overlapping regions of “A1G vs. GC” and “MG vs. GC” was 150, indicating that 150 genes were significantly differentially expressed between not only A1G and GC, but also MG and GC. (**b**) Volcano plot showing the log2 scaled fold change (x-axis) and the minus log10 *p*-value (y-axis) of each gene in the differential expression analysis. Genes with a significant expression change are highlighted as red dots. Prominent transcripts are labelled by GeneSymbol. (**c**) Heatmap showing the gene expression values (normalized as transcripts per millions) of selected genes in each sample, significantly differentially expressed between any two groups. Red and blue color intensity correlates with relative signal abundance of selected genes. (**d**) Principal component analysis (PCA) plot, which shows the two first PCs (principal components) values of each sample. Differentially expressed genes were detected by means of DESeq2^37^ cloud using a Wald test, a parametric fit type, and setting an adjusted *p*-value cutoff of 0.05 (*n *= 3 each). Analysis was conducted in R^31^.

Next, we performed RNA-Seq analysis to overview the changes in gene expression of molecules around sulfur compounds. RNA-Seq analysis of the livers of GC, A1G, and MG mice showed that the largest difference in the gene expression profile was between GC and MG mice, suggesting a considerable impact of environmental change consequent to spaceflight (Fig. 3a,b). The number of differentially expressed genes was the highest between GC and MG (Fig. 3a). The volcano plot also showed that differentially expressed genes were abundant with much lower *p*-values in MG vs. GC than in A1G vs. GC and MG vs. A1G (Fig. 3b). The heatmap showed that many genes were differentially expressed between any two of the three groups, especially in MG and GC mice (Fig. 3c). Principal component analysis showed that the gene expression profiles of MG mice were considerably different from those of GC mice, whereas the gene expression profiles in A1G vs. GC and MG vs. A1G were also different (Fig. 3d). Therefore, these results suggest an impact of the space environment on gene expression in the livers of mice, with considerable effects owing to microgravity.

We then performed pathway data prediction using databases, such as Gene Ontology (GO), Reactome, and KEGG pathway (Supplementary Figs. S3, S4 and S5 online). In general, lipid metabolism was in a prominent change between MG and GC mice, as described before^1^. Notably, pathways including biological oxidation, oxidoreductase activity, and glutathione metabolism were among the top ten pathways that largely changed in MG compared to GC mice (Supplementary Fig. S3 online). Furthermore, pathways related to oxidative stress were also changed in A1G vs. GC mice and MG vs. A1G mice, implying that oxidative stress was induced by both the spaceflight environment and microgravity. (Supplementary Figs. S4 and S5 online).

**Figure 4.**
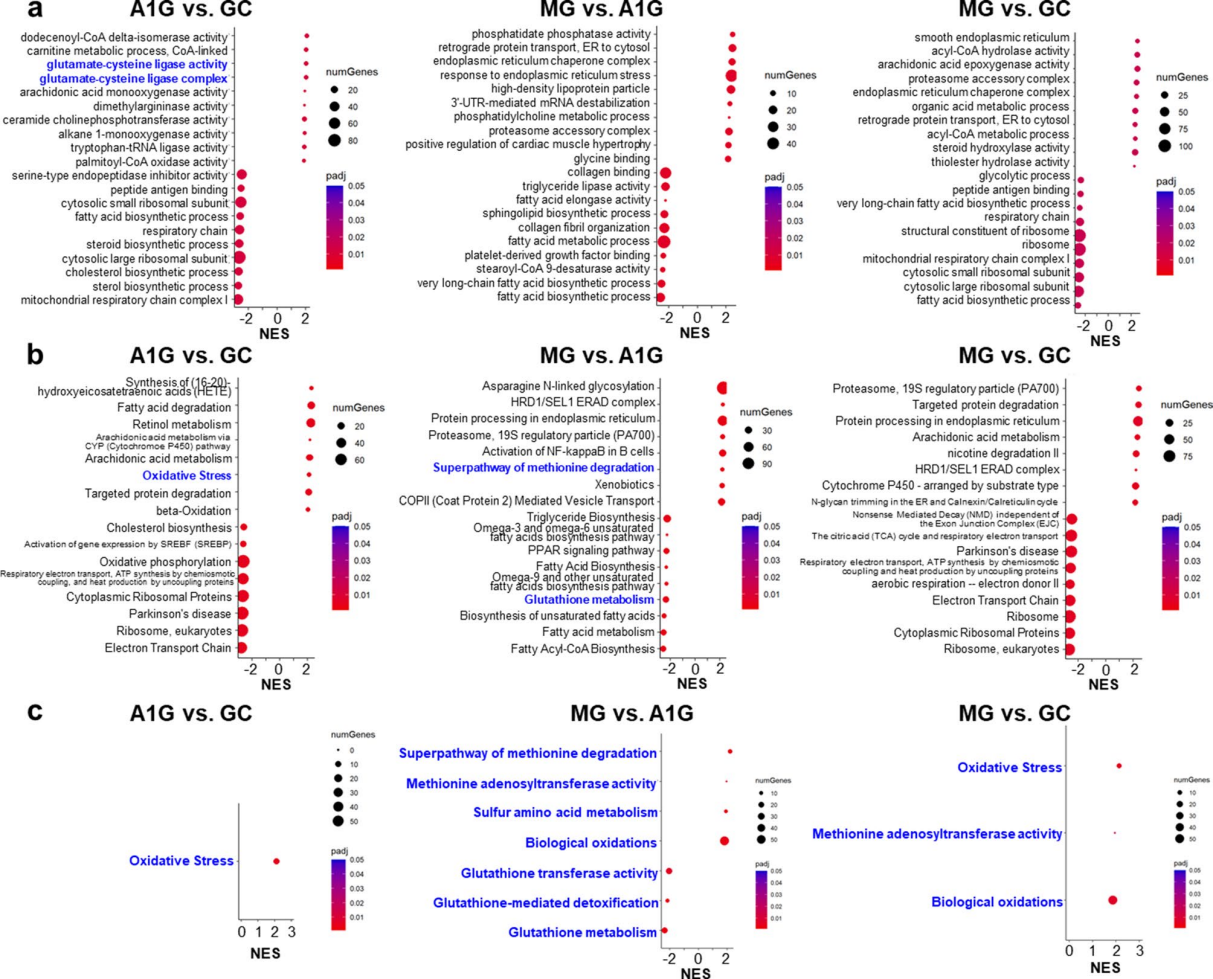
Gene set enrichment analysis (GSEA) of differentially expressed genes in the livers after spaceflight. The normalized enrichment score (NES) of each term is plotted. GSEA of differentially expressed genes annotated to Gene Ontology (GO) (**a**), and Reactome, KEGG, WikiPathways, BIOCYC, and LIPID MAPS (**b**) in the livers compared in between GC, A1G, and MG mice. The top 10 pathways with the highest and lowest NES values were shown each. (**c**) GSEA results about pathways related to oxidative stress, sulfur metabolism, or glutathione metabolism. The dot color, the adjusted p-value of enrichment; NumGenes, the leading-edge genes that drive the enrichment of each category. Pathways colored blue are related to oxidative stress, sulfur metabolism, or glutathione metabolism. Analysis was conducted in R^31^, using the R packages GOseq^38^ and fgsea^39^.

We also performed gene set enrichment analysis (GSEA) of differentially expressed genes. Importantly, changes in some pathways related to oxidative stress or sulfur metabolism were detected in various databases (Fig. 4, Supplementary Fig. S6a and Supplementary Table 2 online). Notably, GSEA revealed that gene sets of ‘oxidative stress’ and ‘biological oxidations’ were upregulated in A1G mice compared to GC mice, as well as in MG mice compared to GC mice (Fig. 4c and Supplementary Fig. S6b online), consistent with the upregulation of gene expression related to these gene sets (Supplementary Table 3 online). These results suggest that both microgravity and other spaceflight environments (e.g., radiation and stressors) change gene expression related to oxidative stress. In contrast, GSEA revealed that gene sets of ‘glutathione transferase activity’, ‘glutathione-mediated detoxification’, and ‘glutathione metabolism’ were downregulated in MG mice compared to A1G mice (Fig. 4c and Supplementary Fig. S6b online), consistent with the downregulation of gene expression related to these gene sets (Supplementary Table 4 online). These results imply that the reducing ability of GSH is lower in MG mice than in A1G mice. Furthermore, GSEA revealed that gene sets of ‘methionine adenosyltransferase (MAT) activity’, ‘superpathway of methionine degradation’, and ‘sulfur amino acid metabolism’ were upregulated in the livers of MG mice compared to A1G mice (Fig. 4c and Supplementary Fig. S6b online), consistent with the upregulation of gene expression related to these gene sets (Supplementary Table 5 online). This upregulated metabolism (production and degradation) of sulfur compounds was considered to be caused by a compensatory metabolism in response to the decrease in the amount of SAM and its derivative cystathionine in MG mice compared to A1G mice (Figs. 1a and 2c).

**Figure 5.**
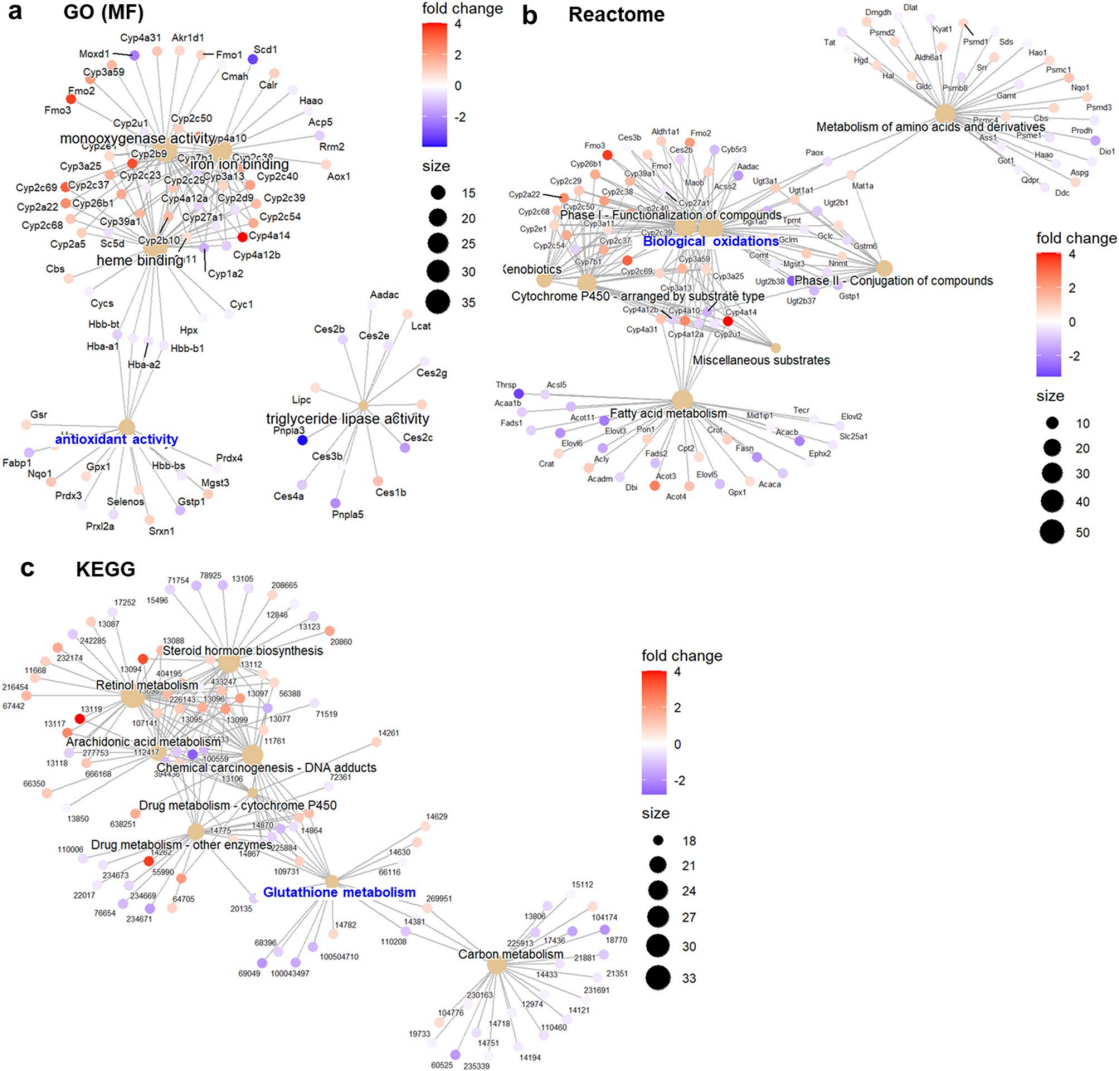
The linkages of genes and biological concepts changed in the livers of MG mice compared to GC mice. GC, control mice on Earth; MG, mice under microgravity in space. Biological concepts were from Gene Ontology (GO) terms (**a**), Reactome (**b**), or KEGG (**c**) pathways as a network. Dot color, the fold change of enrichment in the livers of MG mice compared to GC mice; Size, the node size. Pathways colored blue are related to oxidative stress. Analysis was conducted in R^31^, using the R package clusterProfiler^41^.

Moreover, we investigated the linkages of genes and biological concepts, which suggests that pathways, such as biological oxidations, antioxidant activity, and glutathione metabolism, were central hubs linked to other pathways changed in the livers after spaceflight (Fig. 5 and Supplementary Figs. S7, S8, and S9 online). In particular, there was a strong increase in many subtypes of *CYP* genes, each of which encodes a member of the cytochrome P450 superfamily of enzymes. ROS can be generated while cytochrome P450 catalyzes the oxygenation of an organic substrate^40^. Therefore, *CYP* genes can be key genes related to oxidative stress after spaceflight.

**Figure 6.**
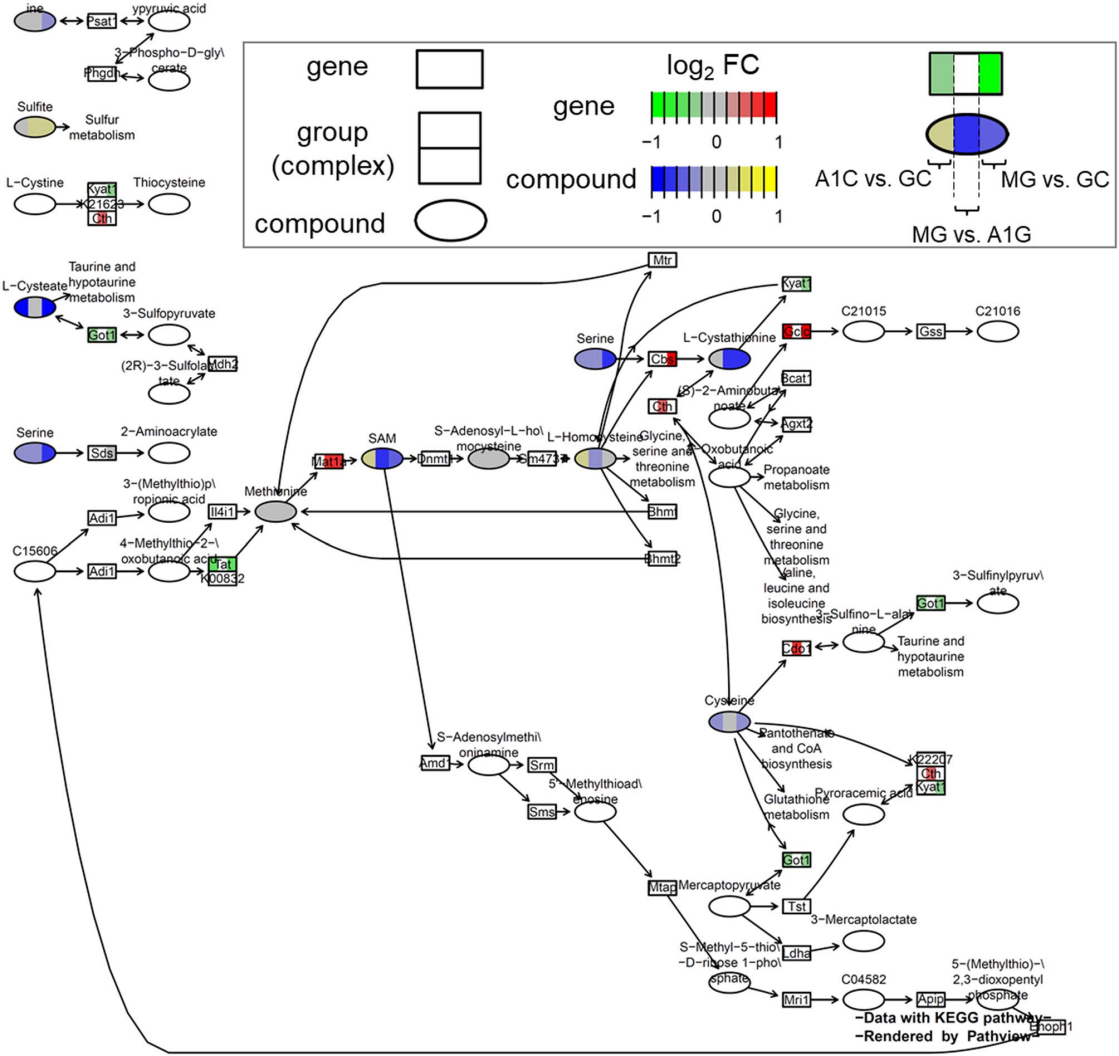
Pathway-based data integration and visualization of KEGG PATHWAY 00270 “Cysteine and methionine metabolism”. Analysis was conducted in R^31^ using the R package pathview^42^, which renders the metabolome and transcriptome data on the pathway. The color of genes and compounds means log_2_ fold change (log_2_FC), separated by three parts containing the ratio of A1G vs. GC (left), MG vs. A1G (middle), and MG vs. GC (right). Control mice on Earth (GC), mice under artificial earth-gravity in space (A1G), and mice under microgravity in space (MG). Images were obtained by KEGG^43,44^.

Finally, we integrated the metabolome and transcriptome data on the important pathways, such as ‘cysteine and methionine metabolism’ and ‘glutathione metabolism’ (Fig. 6 and Supplementary Fig. S10 online). This visualization supports our understanding that spaceflight decreased sulfur-containing compounds in mouse livers with compensatory changes in gene expression, and that artificial gravity mitigated this decrease to some extent. For example, in MG compared to A1G mice, there was a decrease in the volume of SAM, l-homocysteine, serine, l-cystathionine (the middle area of each compound is shown in blue), which means that artificial gravity mitigated the decrease. The upregulation of *Mat1a*, *Cth*, and *Cdo1* genes in MG compared to A1G mice (the middle area of each gene is shown in red) seemed to compensate for the decrease in these sulfur compounds (Fig. 6). On the other hand, spaceflight decreased GSH in the liver (the right area of GSH is shown in blue), but artificial gravity had a small effect on the glutathione pathway (the middle area of GSH and each gene is gray). *Gclc* and *Gst* genes were upregulated in MG compared to GC mice (the right area of each gene is shown in red), which also seemed to compensate for the decrease in GSH (Supplementary Fig. S10 online).

## Discussion

In this study, we demonstrated that compared to ground controls, mice after spaceflight had a lower antioxidant capacity owing to decreased sulfur-containing compounds such as ergothioneine, cysteine, and GSH in the liver, and that the spaceflight environment upregulated gene sets related to oxidative stress and sulfur metabolism, and downregulated gene sets related to GSH activity, whereas artificial earth gravity mitigated these changes in sulfur compounds and gene expression to some extent.

Multivariate analysis of sulfur metabolomics showed that the sulfur profiles of A1G and MG mice were considerably different from those of GC mice, in which cysteine, GSH, and GSSG showed strong correlation coefficients. Specifically, the amount of reduced sulfur compounds (ergothioneine, GSH, cysteine, taurine, thiamine, etc.) and their intermediates (cysteine sulfonic acid, hercynine, *N*-acethylserine, serine, etc.) in the livers of A1G and MG mice were lower than those of GC mice. We also found that some sulfur compounds (SAM, cystathionine, hypotaurine, hercynine, histidine, and *N*-acetylserine) were downregulated only in MG mice compared to GC or A1G mice. These results suggest that the spaceflight environment decreased the amount of sulfur-containing compounds, in which microgravity contributed to the decrease to some extent. In addition, SAM, homocysteine, S-methyl-cysteine, and hypotaurine appeared higher (not significantly) in only A1G than in GC mice. Changes in the expression of gene sets related to GSH and sulfur metabolism in the livers of mice exposed to a space environment have been detected previously using transcriptomic analyses^1,6–8^; however, to the best of our knowledge, this is the first report of measuring the amount of sulfur metabolites in the mouse liver after spaceflight using an LC–MS/MS system. A decrease in sulfur compounds in the liver is important because impairment of sulfur metabolism is strongly associated with chronic liver disease and causes the failure of liver regeneration after tissue damage in cultured hepatic cells, mice, and patients^15,45–47^. Conversely, pharmacological modification of abnormality in hepatic metabolism of sulfur-containing compounds has been effective against damage in cultured hepatic cells and liver injury in rodents^48–51^. Therefore, our results suggest that liver damage after spaceflight could be exacerbated by a decrease in sulfur-containing compounds, which would be alleviated by treatment for sulfur compound imbalance; for example, the intake of these sulfur compounds during spaceflight would be a good strategy. In particular, intake of GSH, ergothioneine, and taurine would be effective against liver damage after spaceflight, because their oral administration has been shown to be protective against liver damage in vivo^19^ and hepatic metabolism in humans^52^.

RNA-Seq analysis of the liver showed that the largest difference in gene expression profiles was between GC and MG mice, suggesting an impact of environmental change and long-term microgravity consequent to spaceflight. In addition, there was a considerable difference in gene expression in the liver between A1G and MG, as has also been shown using RNA-Seq in other organs, such as the spleen and the tyumus^25,26^. Specifically, pathway analyses showed the upregulation of gene sets related to oxidative stress and sulfur metabolism, and the downregulation of gene sets related to GSH activity in the livers of MG mice compared to GC mice, whereas these changes were mitigated in A1G mice to some extent. These results suggest that both microgravity and other spaceflight environments (e.g., radiation and stressors) changed gene expression. However, the RNA-seq results, which were extensively discussed in this study, suffer from the limitation of having been performed with only *n* = 3.

In a project conducted by JAXA using mice in space using an MHU, a unique attempt was made to create an artificial earth gravity group (A1G) during spaceflight using centrifugation cages^24^. However, to date, no study has investigated the influence of long-term microgravity on the liver using A1G mice. Notably, our experiments revealed that centrifuging at 1 g alleviated the decrease in sulfur-containing compounds, upregulation of gene sets related to oxidative stress and sulfur metabolism, and downregulation of gene sets related to GSH activity in the liver after spaceflight, suggesting that long-term microgravity contributes to the imbalance in sulfur metabolomics. Changes in the organs between A1G and MG were also observed in femur bone density and soleus/gastrocnemius muscle mass, spleen, retina, and thymus^24–26,28^, suggesting that in-flight artificial gravity can mitigate some of the harmful effects of microgravity during spaceflight. Although these changes in the other organs due to microgravity may have indirectly influenced the livers of MG mice, unidentified effects by microgravity may also have directly or indirectly affected the oxidative state and gene expression in the liver.

The conditional difference between A1G and GC mice is a spaceflight environment other than microgravity. As to exposure to radiation in space, total radiation during spaceflight was 0.26 ± 0.01 mGy/day and the dose equivalent rate was 0.53 ± 0.04 mSv/day in this project (MHU-2)^26^. Sulfur-containing compounds play a role in defense against oxidative stress induced by radiation in the liver^4^, and radiation during spaceflight may have decreased the reduced form of sulfur-containing compounds. It should also be noted that both MG and A1G mice experienced hypergravity during launching and landing. Moreover, it is inevitable that A1G mice experienced microgravity during transfers from the launch rocket to the ISS and back because there was no centrifuge inside the rocket^24^. Thus, the impact of these events on redox reactions and gene expression should be considered. In addition to these concerns, the transportation period from landing to dissection of mice may also have affected the data^24^. Taken together, the conditional difference between A1G and GC mice includes physical stress owing to launching and landing (hypergravity, microgravity, and transportation), as well as the space environment such as higher radiation.

In this study, for the first time, we observed a decrease in sulfur compounds in the livers of mice after spaceflight. A decrease in sulfur compounds, which are antioxidants against the oxidative stress induced by the stressful space environment, may contribute to the development of liver damage caused by spaceflight. It may be necessary to reconsider the roles of antioxidants, such as sulfur compounds, to counteract the negative effects of spaceflight, and approaches to maintaining homeostasis in hepatic sulfur metabolism can be a promising target in the therapy of liver disease during or after spaceflight. In conclusion, our data suggest that mice after spaceflight have less antioxidant capacity with a decrease in sulfur-containing compounds in the liver.

## Methods

### Animal samples

All mouse experiments were approved by the Institutional Animal Care and Use Committee of the University of Tsukuba, JAXA, Explore Biolabs, and NASA, and were conducted in accordance with the applicable guidelines in Japan and the United States of America. All animal procedures conformed to the ARRIVE guidelines for reporting animal research^53^. The mouse liver samples were unanalyzed samples from the mouse breeding experiments performed by JAXA on the International Space Station (ISS) Kibo in 2017 as described previously^23^. Briefly, pre-launch acclimation was conducted in the Space Station Processing Facility Science Annex at the Kennedy Space Center (KSC) (FL, USA). Five-week-old C57BL/6J male mice (stock number 000664) were purchased from The Jackson Laboratory (Bar Harbor, ME, USA) for space and ground control experiments. Twelve flight mice were given a health check by the NASA Launch Facility veterinarian before installation into the transportation cage unit (TCU). The selected mice were installed into the TCU and launched to the ISS via Space-X12 on August 14, 2017 (GMT). The Space-X12 arrived at the ISS on August 17, 2017 (GMT), and mice were relocated from the TCU to the habitat cage units (HCU) by an astronaut. We divided the mice into two groups of six mice each [AG (≈ 1 g, at a centrifugation speed of 77 rpm) and MG], and they were caged in the ISS for approximately 30 days. This time span was determined based on the flight schedule of this mission (mission #: MHU-2). The mice were relocated from the HCU to the TCU for return to Earth on September 16, 2017 (GMT), and the Dragon vehicle loading the TCU splashed down in the Pacific Ocean off the coast of California on September 17, 2017 (GMT). All the mice were sacrificed and dissected within 36.5 h of splashdown. Isoflurane-anesthetised mice were euthanized by exsanguination, and the organs were removed and snap-frozen in liquid N_2_, kept in a − 80 °C freezer^26^. A ground control experiment was conducted from January 18 to March 22, 2018, at JAXA (Tsukuba, Japan). The environmental conditions of housing were as close as possible to acclimation conditions at KSC, and mouse diets and water consumption were the same as those used during the flight as previously described; for example, the temperature was controlled at 23 ± 3 °C in flight and 23.0 °C on the ground, the humidity was controlled at 40 ± 15% in flight and 47.3% on the ground, and the CO_2_ concentration was 0.29% for A1G mice onboard, 0.33% for MG mice onboard, and 0.04% on the ground^23,26^.

### Sulfur metabolomics

Sulfur metabolomics was performed using the Sulfur Index service in Japan with an LC–MS/MS system. Recently, the measurements of reactive sulfur compounds have been broken through by the multiple-reaction monitoring mode (MRM) of mass spectrometry using alkylating reagents such as monobromobimane^20^. We named this method the Sulfur Index as the sulfur metabolome^54–57^. The 3 ml extraction reagent was prepared as follows; 25 µl of 2.5 mM D-camphor-10-sulfonic acid sodium salt (CSA, internal standard), 25 µl of 100 mM monobromobimane (mBBr; Thermo Fisher SCIENTIFIC), 25 µl of 200 mM Tris–HCl (pH8.8), 0.55 ml of Milli-Q water, and 2.375 ml of methanol were mixed. Each liver sample was cut into ~ 10 mg on dry ice. 120 µl of the prepared extract reagent was added to the ~ 10 mg of liver, and then immediately homogenized with a pestle on ice. After centrifugation, 90 µL of the supernatant was used as the sample for mass spectrometry (LC–MS/MS). The mBBr reagent was used as a thiol group-specific alkylation reagent, which means that it reacts with compounds, including free SH residues. Thus, both bimanyl and non-bimanyl compound were present in the final sample. Bimanyl compounds are stable from chemical reactions, and non-bimanyl sulfur compounds are primarily not active, which means that this sulfur index method not only avoids the autooxidation that occurs during the experimental sample preparation process, but is also feasible for taking snapshots of the liver’s physiological redox state. Therefore, this sulfur index is a specialized method for the visualization of oxidative stress by measuring whether the sulfur compound in the sample is detected in the oxidized or reduced form. The target metabolite levels were determined from the peak area by mass chromatography and represented as relative amounts after normalization with the peak area of the internal standard (d-camphor-10-sulfonic acid). The MS signal was identified and quantified manually and corrected by the weight of the liver subjected to sample preparation (*n* = 6 each). The values were then normalized, as shown in the legend of each graph. We also performed a multivariate sample-to-sample similarity mapping analysis based on the detected sulfur-related compound data using the R software vegan package.

### RNA extraction

A piece of liver weighing 20–30 mg was cut from each liver sample on dry ice. Homogenization was performed using a disposable homogenizer (Biomasher II, AS ONE CORPORATION, Osaka, Japan) on ice. RNA was isolated and purified using NucleoSpin RNA (Takara, Kusatsu, Shiga, Japan) according to the manufacturer's instructions. RNA was eluted in 40 μl RNase-free water per sample. The concentration and absorbance ratios of all RNA samples were measured using a NanoDrop 2000 spectrophotometer (Thermo Fisher Scientific, Waltham, MA, USA).

### RNA next-generation sequencing

The messenger RNA (mRNA) obtained from the livers of mice was sequenced using NovaSeq 6000 (Illumina Inc., San Diego, CA). We used the Poly(A) mRNA Magnetic Isolation Module (New England Biolabs, Ipswich, MA, USA) for poly(A) RNA preparation, and NEBNext Ultra Directional RNA Library Prep Kit for Illumina (New England Biolabs, Ipswich, MA, USA) for library preparation. These data were analyzed with the RaNA-Seq^58^ cloud platform for the rapid analysis and visualization of RNA-Seq data to perform FASTQ preprocessing (fastp 0.19.4) and quantification (salmon 0.9.1) of the samples. Total reads obtained for each library ranged between 18 and 26 million, and properly matched reads/read pairs were between 81 and 94%. Differentially expressed genes were detected by means of DESeq2^37^ cloud using a Wald test, a parametric fit type, and setting an adjusted *p*-value cutoff of 0.05.

## Supplementary Information

Supplementary Information.
