# Supplementary material for: Impact of spaceflight and artificial gravity on sulfur metabolism in mouse liver: sulfur metabolomic and transcriptomic analysis
Source: Sci Rep. 2021 Nov 8;11:21786. doi: 10.1038/s41598-021-01129-1 (PMC8575787; doi:10.1038/s41598-021-01129-1)
Supplement: Supplementary file 1 — Supplementary Information. [file 41598_2021_1129_MOESM1_ESM.pdf]

Impact of spaceflight and artificial gravity on sulfur metabolism in mouse liver: sulfur metabolomic and transcriptomic analysis

Ryo Kurosawa[1,2,*], Ryota Sugimoto[1], Hiroe Imai[3], Kohei Atsuji[1,2], Koji Yamada[1,2], Yusuke Kawano[1,4], Iwao Ohtsu[1,4,+,*] & Kengo Suzuki[1,2,5,+,*]

[1]euglena Co., Ltd., Tokyo, Japan

[2]Microalgae Production Control Technology Laboratory, RIKEN, Kanagawa, Japan

[3]R&D Center for Tailor-Made-QOL, University of Tsukuba, Ibaraki, Japan

[4]Faculty of Life and Environmental Sciences, University of Tsukuba, Ibaraki, Japan

[5]Tohoku University Advanced Graduate Program for Future Medicine and Health Care

*corresponding.author, Kengo Suzuki: suzuki@euglena.jp.

Iwao Ohtsu: ohtsu.iwao.fm@u.tsukuba.ac.jp

Ryo Kurosawa: ryo.kurosawa@riken.jp

+these authors contributed equally to this work

<u>Supplemental Information</u>

- Supplementary Figures (10)
- Supplementary Tables (5)
- Supplementary References

Supplementary Figure S1

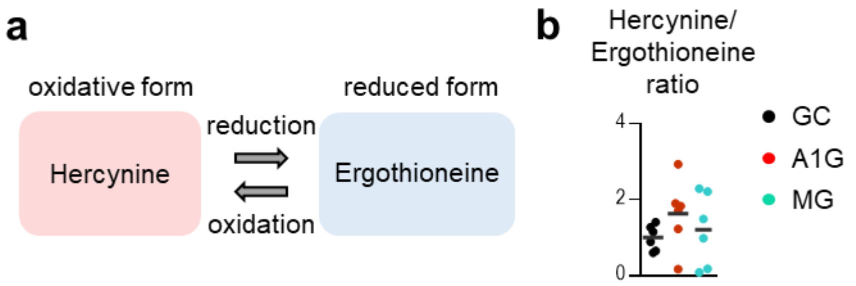

Supplementary Figure S1. Shift of ergothioneine to the oxidized form. (**a**) An illustration of the reduction and oxidization reaction of ergothioneine and hercynine. (**b**) The ratio of the relative amount of hercynine to ergothioneine in the livers of control mice on Earth (GC), mice under artificial earth gravity in space (A1G), and mice under microgravity in space (MG). Each horizontal bar represents the mean. $*p < 0.05$. Comparisons of parameters were performed with one-way ANOVA followed by Dunnett's test for multiple comparisons ($n = 6$ each). Dot plots were made using GraphPad Prism 6.07 for Windows, GraphPad Software, San Diego, CA USA, www.graphpad.com.

Supplementary Figure S2

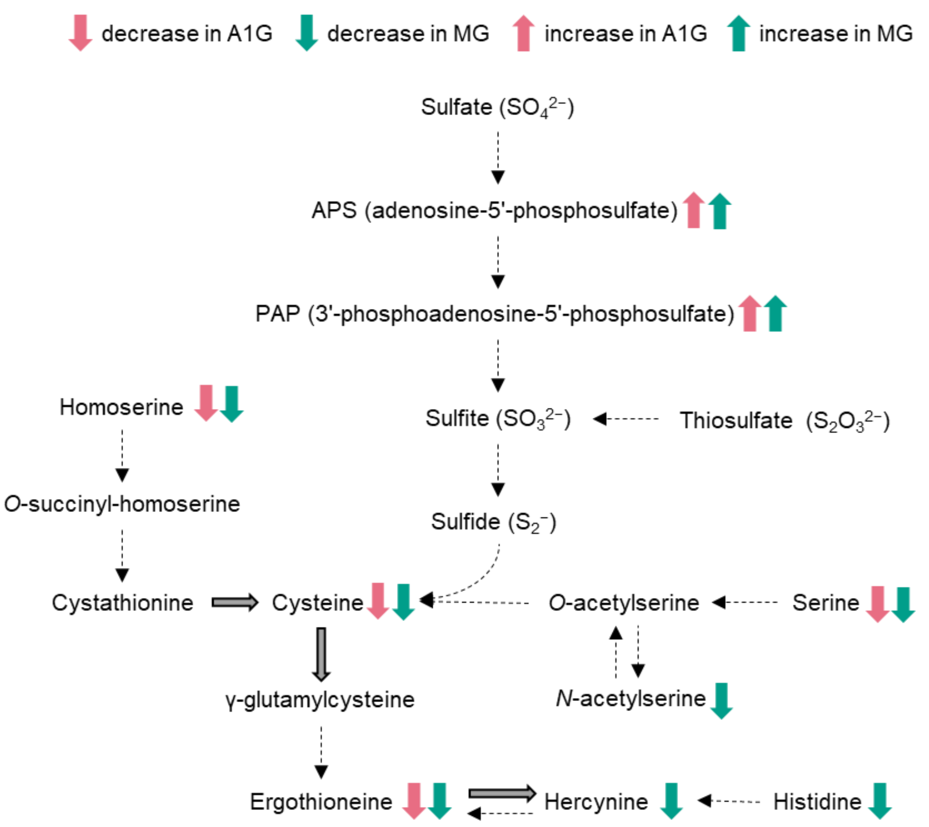

Supplementary Figure S2. An illustration of the metabolic network of sulfur compounds in microorganisms. between compounds indicates metabolic reactions in microorganisms. indicates metabolic reactions in mammals and microorganisms. or indicates a decrease and or indicates an increase in A1G or MG mice, compared to GC mice. GC, control mice on Earth; A1G, mice under artificial earth gravity in space; MG, mice under microgravity in space. Comparisons of parameters were performed with one-way ANOVA followed by Dunnett's test for multiple comparisons (n = 6 each).

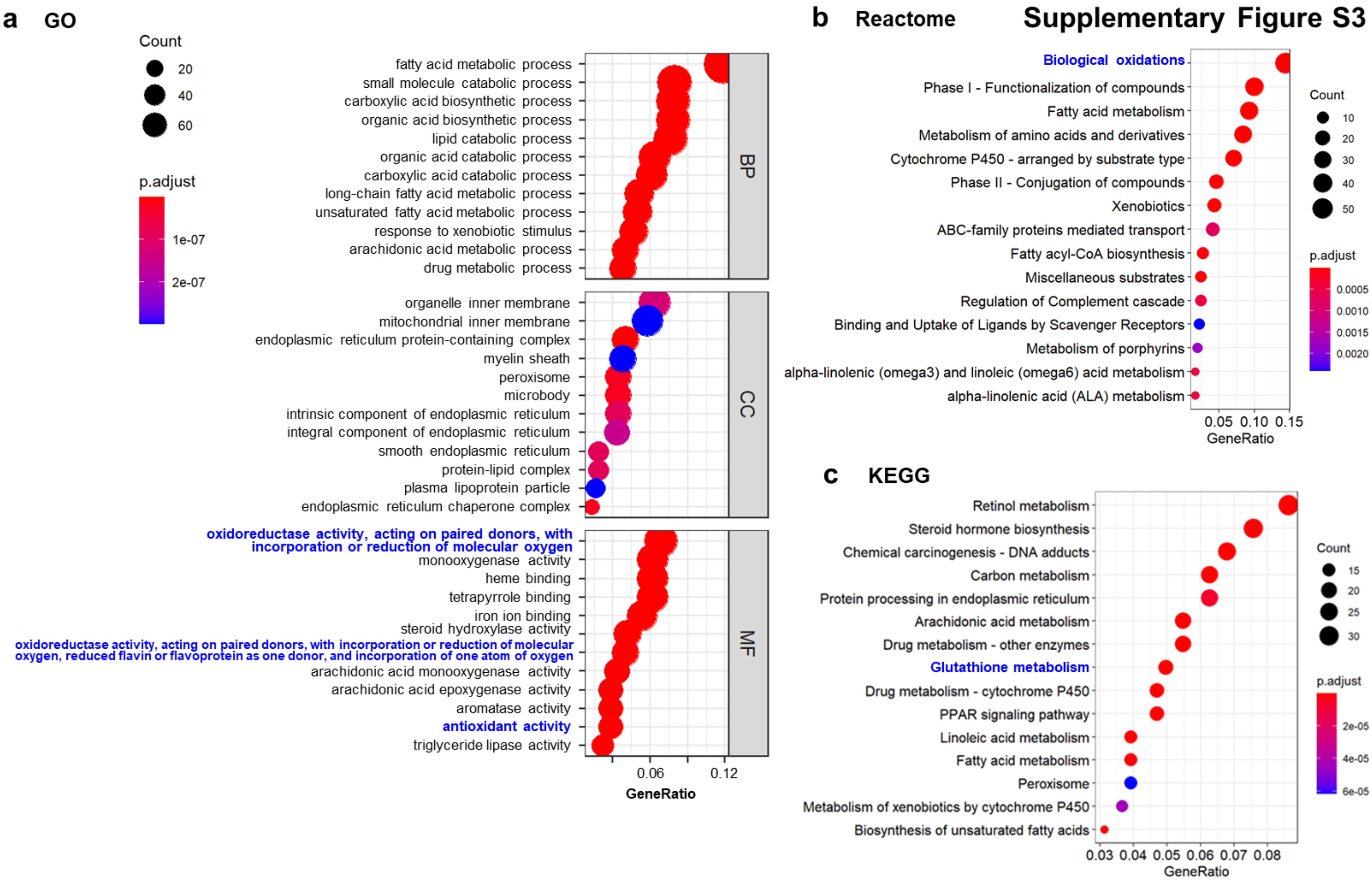

Supplementary Figure S3. Significant changes in genes related to oxidative stress and glutathione metabolism in the livers of MG mice compared to GC mice. GC, control mice on Earth; MG, mice under microgravity in space. Differentially expressed genes in the livers of MG compared to GC mice were annotated to biological concepts. Biological concepts are from GO terms (**a**), Reactome (**b**), or KEGG (**c**) pathways as a network. Dot color indicates the adjusted p-value of enrichment. Count means the number of significant genes in the pathway. Gene ratio means the ratio of Count to the number of genes in the pathway. Pathways colored blue are related to oxidative stress or glutathione metabolism. Analysis was conducted in R[1], using the R package clusterProfiler[2].

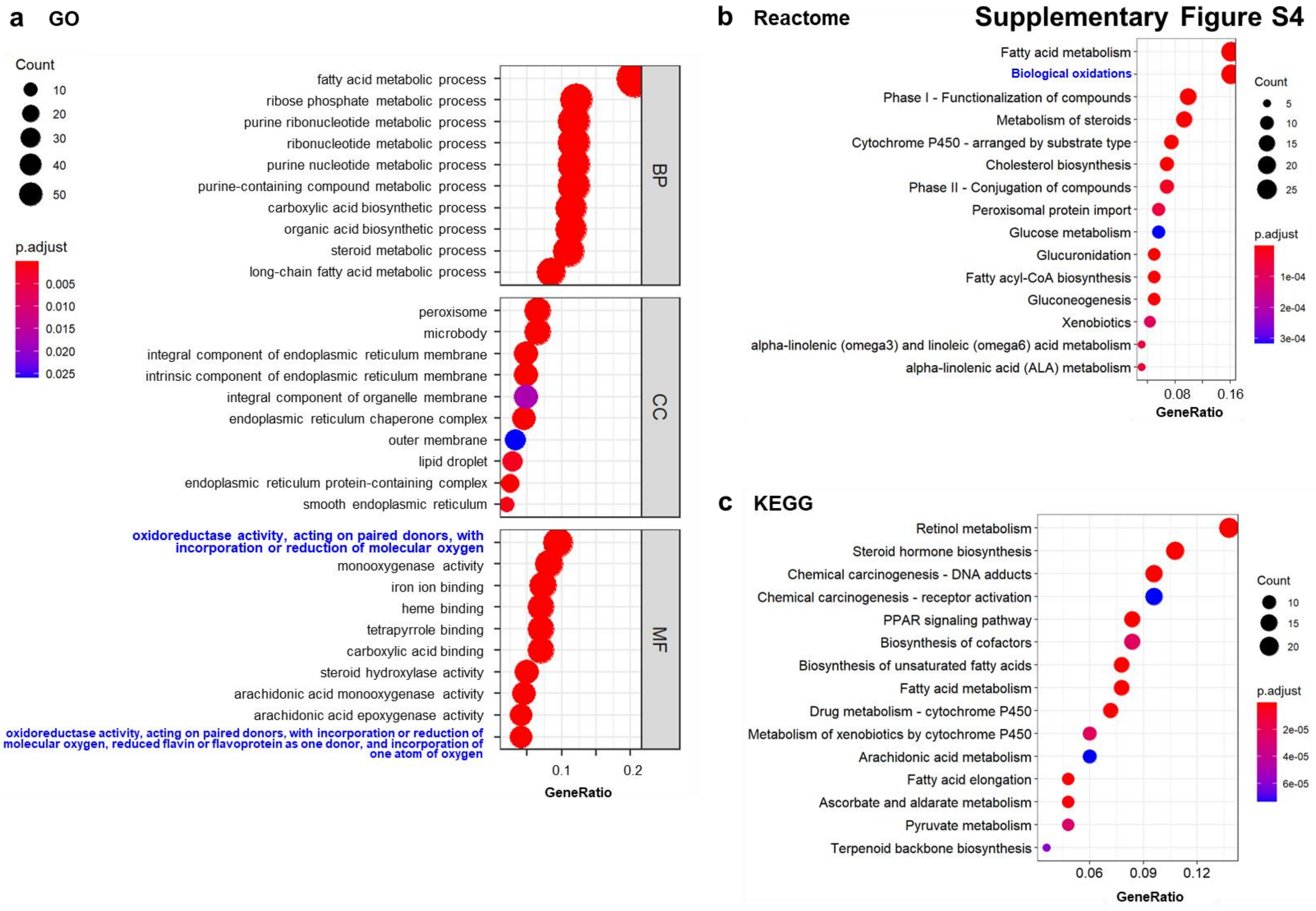

Supplementary Figure S4. Significant changes in genes related to oxidative stress and glutathione metabolism in the livers of A1G mice compared to GC mice. GC, control mice on Earth; A1G, mice under artificial earth gravity in space. Differentially expressed genes in the livers of MG compared to A1G mice were annotated to biological concepts. Biological concepts are from GO terms (**a**), Reactome (**b**), or KEGG (**c**) pathways as a network. Dot color indicates the adjusted p-value of enrichment. Count means the number of significant genes in the pathway. Gene ratio means the ratio of Count to the number of genes in the pathway. Pathways colored blue are related to oxidative stress or glutathione metabolism. Analysis was conducted in R[1], using the R package clusterProfiler[2].

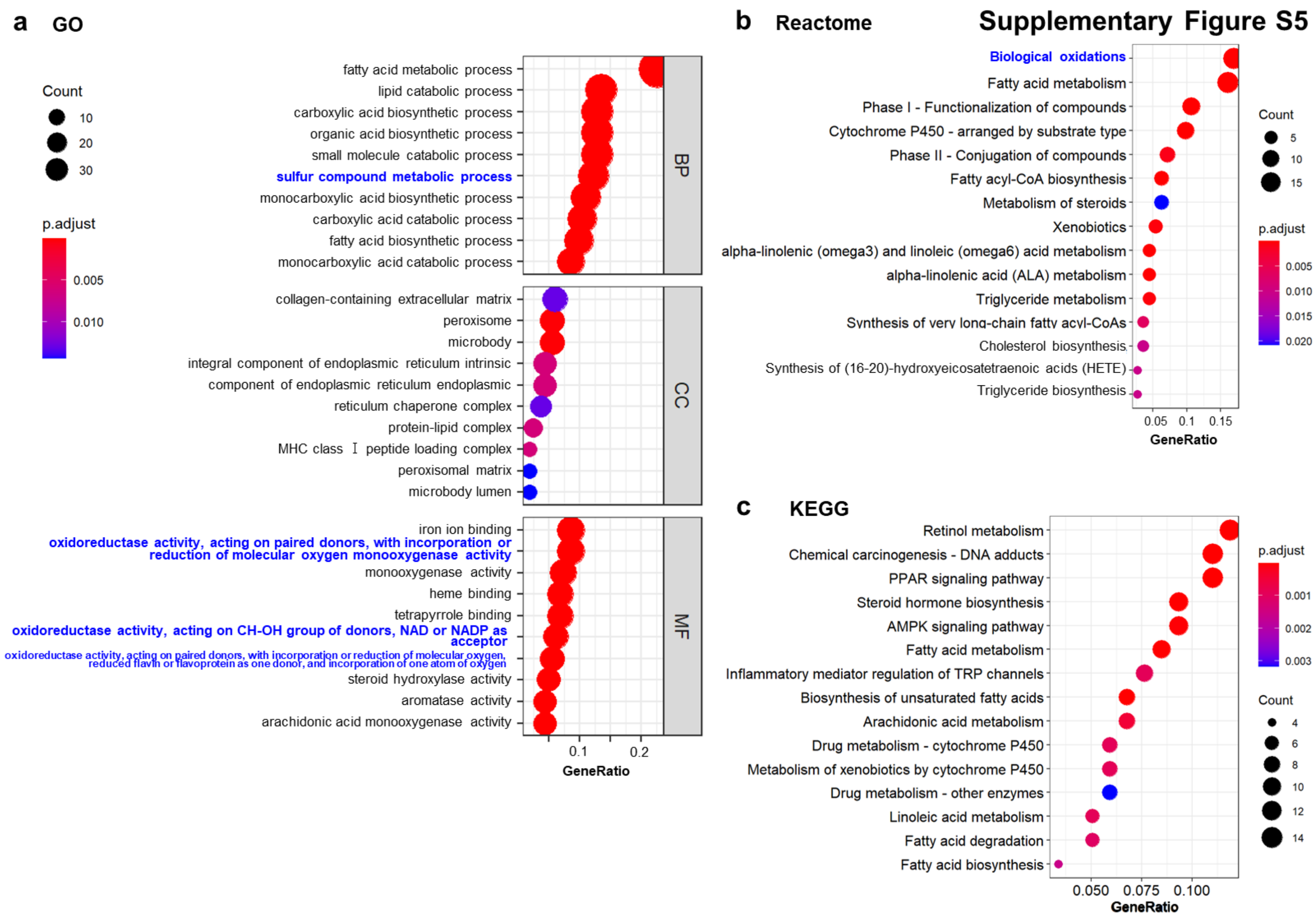

Supplementary Figure S5. Significant changes in genes related to oxidative stress and glutathione metabolism in the livers of MG mice compared to A1G mice. A1G, mice under artificial earth gravity in space; MG, mice under microgravity in space. Differentially expressed genes in the livers of MG compared to A1G mice were annotated to biological concepts. Biological concepts are from GO terms (**a**), Reactome (**b**), or KEGG (**c**) pathways as a network. Dot color indicates the adjusted p-value of enrichment. Count means the number of significant genes in the pathway. Gene ratio means the ratio of Count to the number of genes in the pathway. Pathways colored blue are related to oxidative stress or glutathione metabolism. Analysis was conducted in R[1], using the R package clusterProfiler[2].

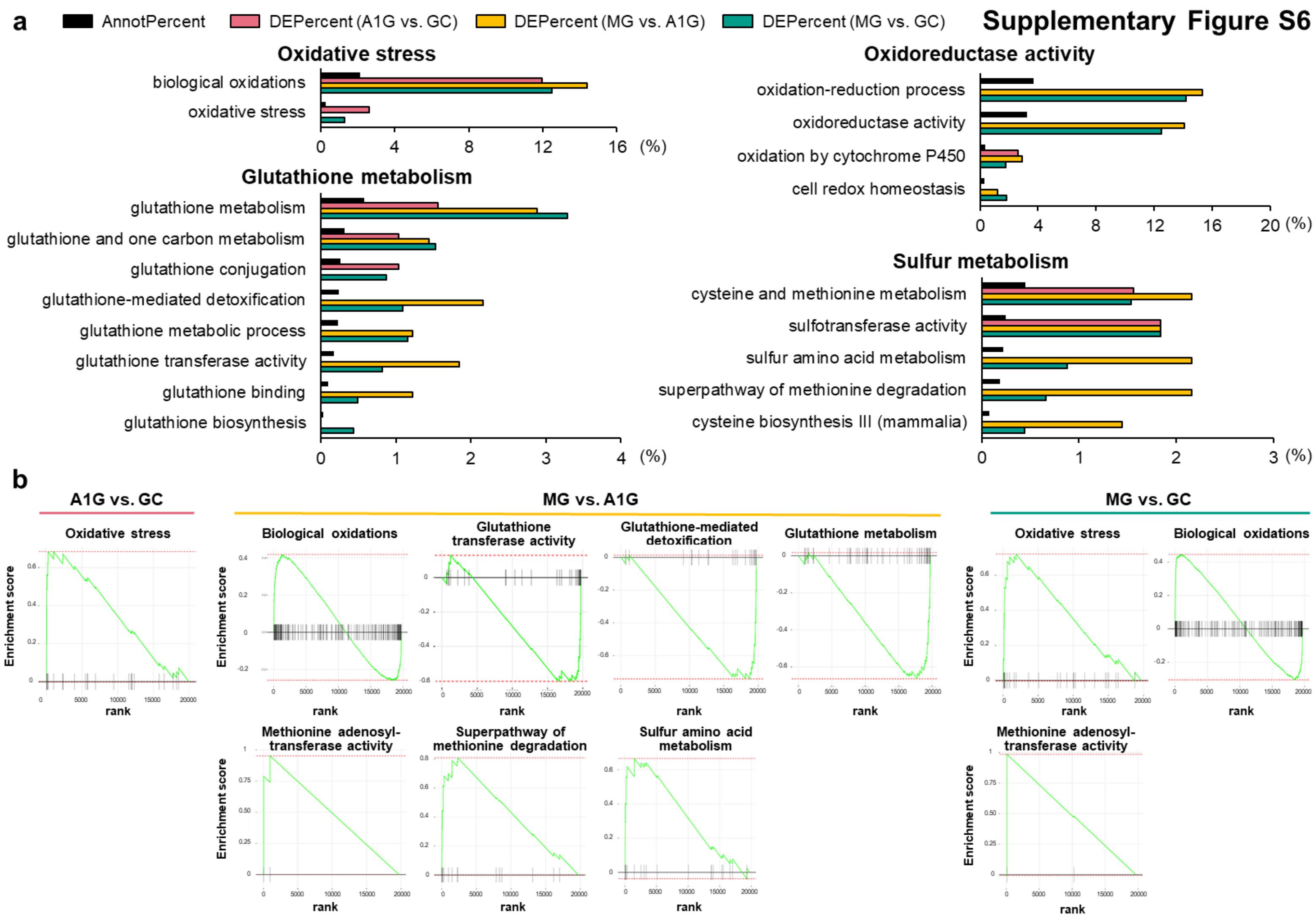

Supplementary Figure S6. Functional enrichment results and gene set enrichment analysis (GSEA) for the data of transcriptomics from the livers of control mice on Earth (GC), mice under artificial earth gravity in space (A1G), and mice under microgravity in space (MG). (**a**) Functional enrichment results with Gene Ontology (GO) pathways. The black column shows the percentage of genes annotated to each term (AnotPercent, the ratio of the number of genes annotated to each GO term to the number of genes annotated to GO). Color columns show the percentage of differentially expressed genes annotated to each GO term (DEPercent, the ratio of the number of differentially expressed genes annotated to each GO term to the total number of genes annotated to each GO term) between two groups. (**b**) GSEA with GO terms and pathways. RUG plots showing the enrichment score against the gene rank. Genes annotated to each category are displayed as marks along the x-axis. Analysis was conducted in R[1], using the R packages GOseq[3] and fgsea[4].

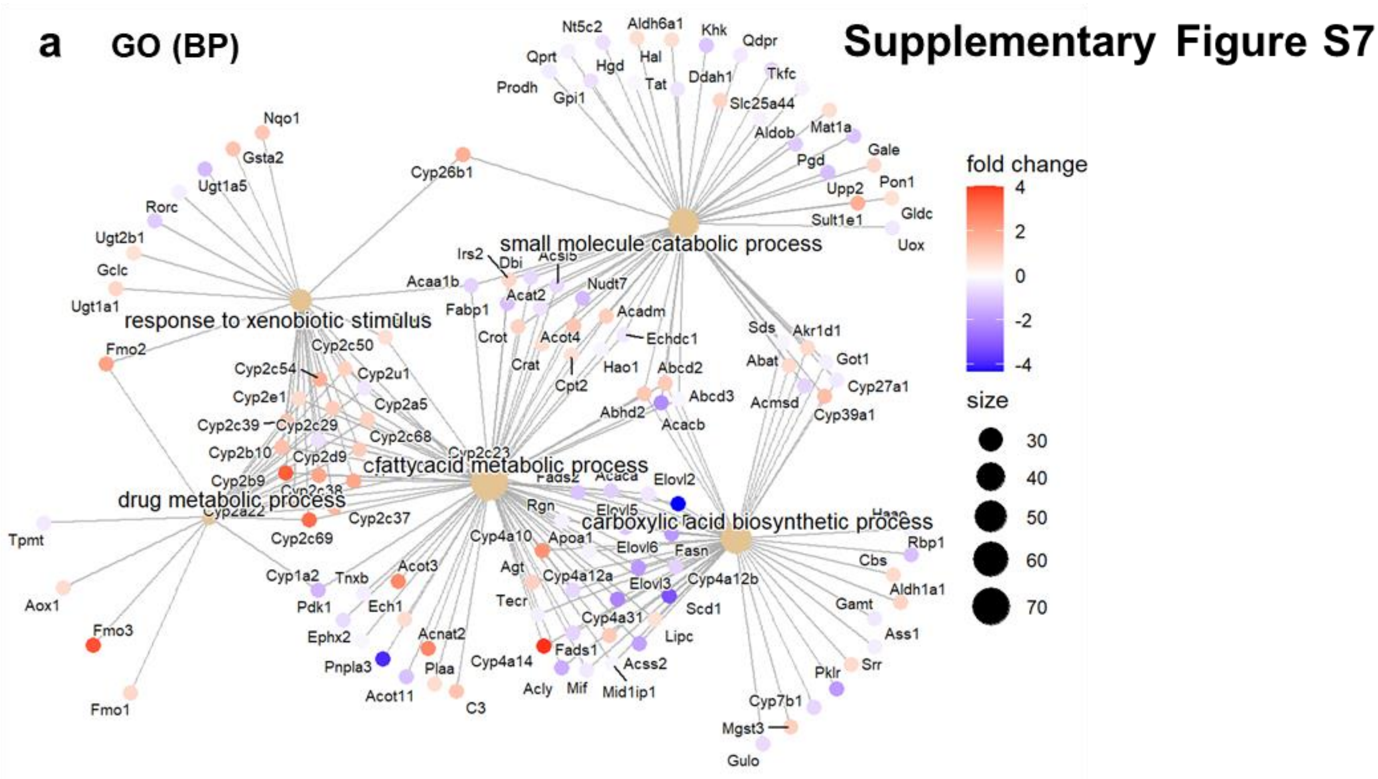

Supplementary Figure S7. The linkages of genes and biological concepts changed in the livers of MG mice compared to GC mice. GC, control mice on Earth; MG, mice under microgravity in space. (**a**) Biological concepts was from GO terms as a network. Dot color indicates the fold change of enrichment in the livers of MG compared to GC mice. "Size" means the node size. Analysis was conducted in R[1], using the R package clusterProfiler[2].

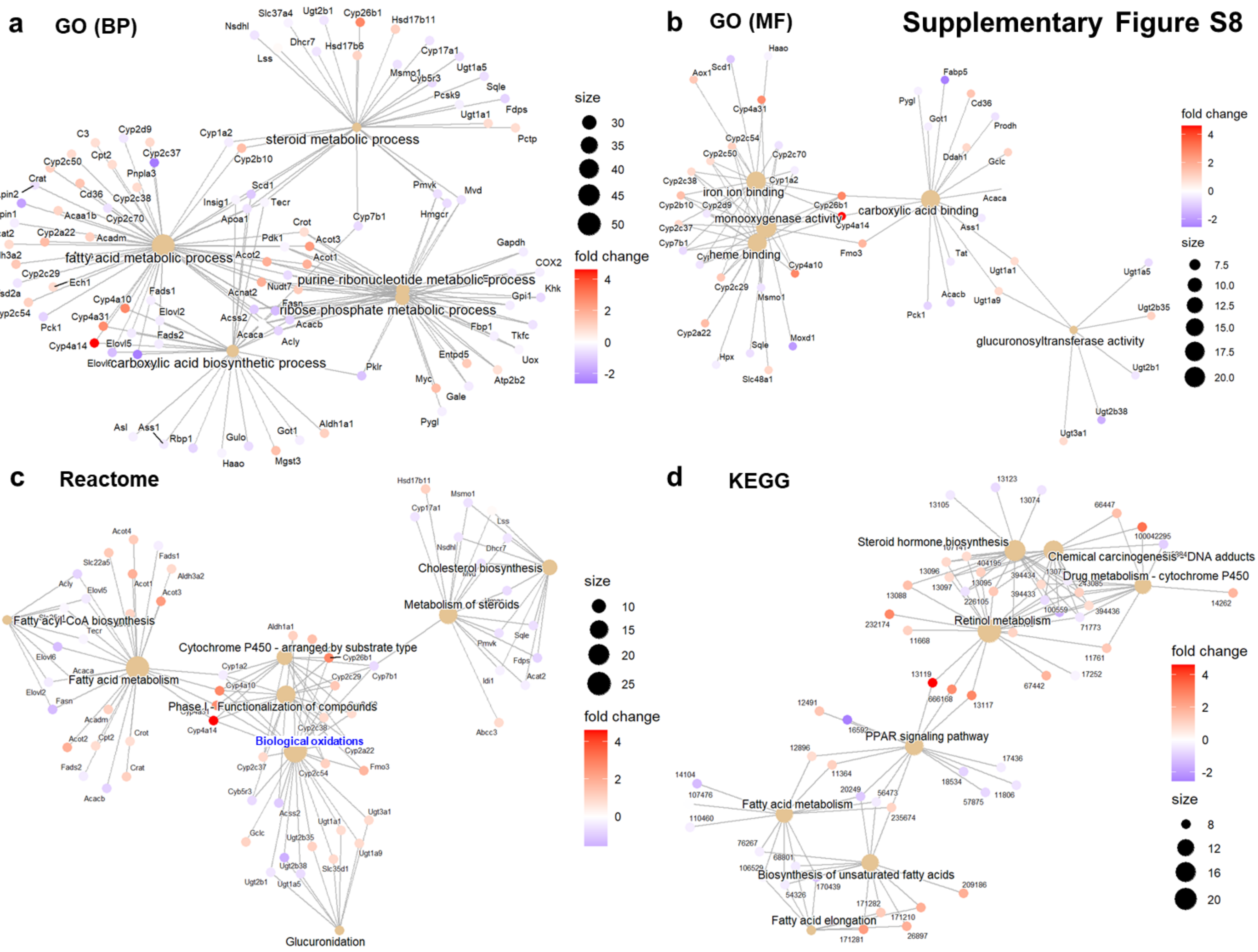

Supplementary Figure S8. The linkages of genes and biological concepts changed in the livers of A1G mice compared to GC mice. GC, control mice on Earth; A1G, mice under artificial earth gravity in space. Biological concepts were from GO terms (**a, b, and c**), or KEGG pathways (**d**) as a network. Dot color indicates the fold change of enrichment in the livers of A1G compared to GC mice. “Size” means the node size. Analysis was conducted in R[1], using the R package clusterProfiler[2].

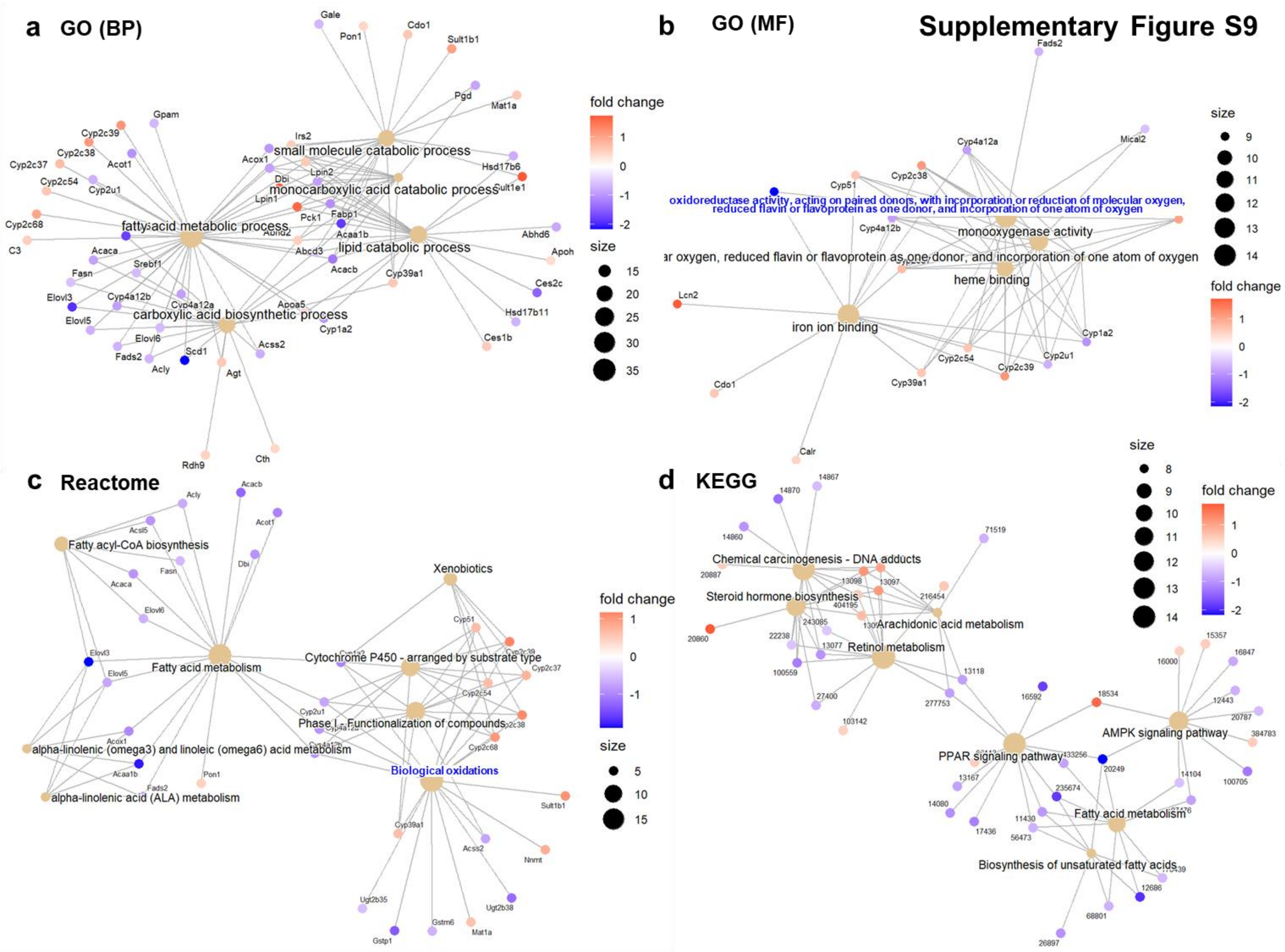

Supplementary Figure S9. The linkages of genes and biological concepts changed in the livers of MG mice compared to A1G mice. A1G, mice under artificial earth gravity in space; MG, mice under microgravity in space. Biological concepts were from GO terms (**a, b, and c**), or KEGG pathways (**d**) as a network. Dot color indicates the fold change of enrichment in the livers of MG compared to A1G mice. "Size" means the node size. Pathways colored blue are related to oxidative stress. Analysis was conducted in R[1], using the R package clusterProfiler[2].

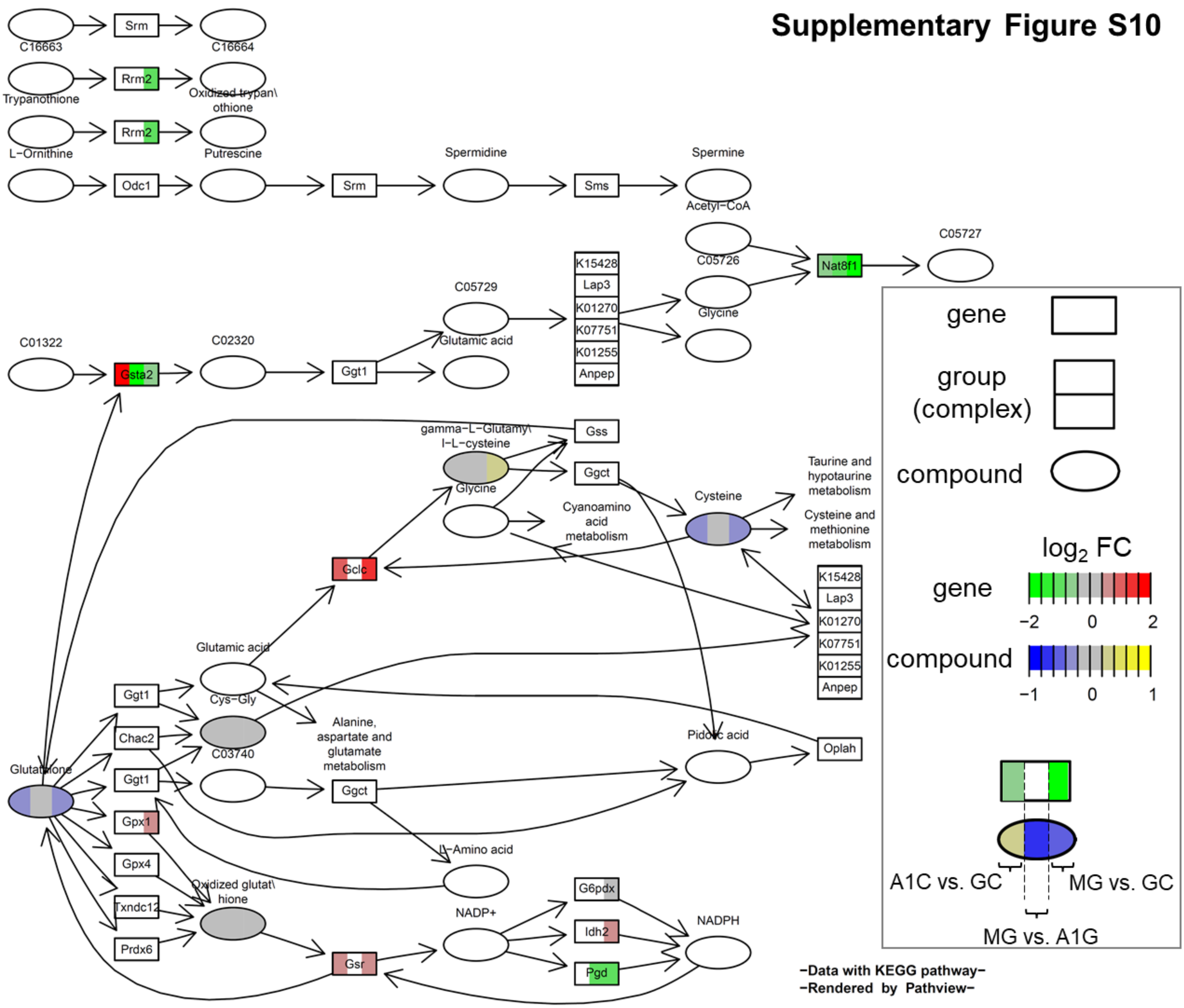

Supplementary Figure S10. Pathway-based data integration and visualization of KEGG PATHWAY 00480 "Glutathione metabolism". Analysis was conducted in R[1] using the R package pathview[5], which renders the metabolome and transcriptome data on the pathway. The color of genes and compounds means $\log_2$ fold change ($\log_2$FC), separated by 3 parts containing the ratio of A1G vs. GC (left), MG vs. A1G (middle), and MG vs. GC (right). Control mice on Earth (GC), mice under artificial earth-gravity in space (A1G), and mice under microgravity in space (MG). Images were obtained by KEGG[6,7].

	PC1	PC2		PC1	PC2
GS-bimane	-0.98798	-0.0501	Cysteine-S-bimane	-0.1589	0.009242
Thiamine	-0.79727	-0.2964	GSSG [M+2H]2+	-0.13726	0.95857
Cysteine-bimane	-0.76822	-0.37685	*O*-phosphoserine	-0.12745	0.004461
Homoserine	-0.71144	0.005384	Glucose	-0.12271	0.146768
Taurine	-0.65587	-0.15588	Urea	-0.12053	0.090003
Histidine	-0.64502	0.203608	Homocysteine - bimane	-0.10626	0.506683
Hercynine	-0.63165	0.374243	GS-S2-SG [M+2H]2+	-0.094	-0.42692
Serine	-0.59173	-0.05081	Hypotaurine	-0.07112	0.119149
N-Acetylserine	-0.53702	-0.02679	Methionine	-0.0672	0.053662
Cysteine sulfonic acid	-0.47438	-0.27639	S-Methyl-cysteine	-0.05995	0.71875
Cystathionine	-0.44682	0.070911	Sulphide-dibimane	0.057161	0.671094
Ergothioneine	-0.44055	-0.64936	Lactic acid	0.147753	0.314428
GS-S2-bimane	-0.37125	-0.13703	γ-glutamylcysteine-bimane	0.27159	-0.4545
GS-S-bimane	-0.23257	0.155242	Sulfite-bimane	0.307009	-0.80244
Thiosulfate-bimane	-0.21526	0.09968	GS-S3-bimane	0.447999	-0.60946
S-Adenosylhomocysteine	-0.19237	0.087156	GS-S3-SG [M+2H]2+	0.45642	-0.58153
S-Adenosylmethionine	-0.17477	0.344367	GS-S4-bimane	0.460724	-0.55129
Cysteineglycicine-bimane	-0.16298	0.087609	PAP	0.515578	0.71932
GS-S-SG [M+2H]2+	-0.16237	0.691534	APS	0.526634	0.657506

Supplementary Table 1. Correlation coefficients derived from a non-metric multidimensional scaling (NMDS) of sulfur metabolomics in the livers of control mice on Earth (GC), mice under artificial earth gravity in space (A1G), and mice under microgravity in space (MG). PC, principal coordinate; GS, glutathione; GSSG, glutathione disulfide; PAP, 3'-phosphoadenosine-5'-phosphosulfate; APS, adenosine-5'-phosphosulfate.

	Pathway ID	Pathway	Database	num Genes	ES	NES	size	*p*-value	*p*adj
**A1G vs. GC**	WP412	oxidative stress	Wiki Pathways	9	0.684	2.105	28	2.1×10^{-4}	5.0×10^{-3}

	GO ID or Pathway ID	GO term Pathway	Category or Database	num Genes	ES	NES	size	*p*- value	*p*adj
**MG vs. A1G**	R-MMU-211859	biological oxidations	REACTOME	55	0.419	1.828	211	2.0×10^{-4}	4.0×10^{-3}
	GO:0004364	glutathione transferase activity	Function	24	-0.603	-2.065	46	2.0×10^{-4}	7.0×10^{-3}
	MOUSE_PWY-4061	glutathione-mediated detoxification	BIOCYC	10	-0.742	-2.172	23	1.9×10^{-4}	4.0×10^{-3}
	mmu00480	glutathione metabolism	KEGG	23	-0.665	-2.365	57	2.0×10^{-4}	4.0×10^{-3}
	GO:0004478	methionine adenosyltransferase activity	Function	6	0.952	1.956	6	2.0×10^{-4}	7.0×10^{-3}
	MOUSE_PWY-5328	superpathway of methionine degradation	BIOCYC	12	0.809	2.217	18	2.0×10^{-4}	4.0×10^{-3}
	R-MMU-1614635	sulfur amino acid metabolism	REACTOME	9	0.666	1.918	22	2.0×10^{-4}	4.0×10^{-3}

	GO ID or Pathway ID	GO term Pathway	Category or Database	num Genes	ES	NES	size	*p*-value	*p*adj
**MG vs. GC**	WP412	oxidative Stress	Wiki Pathways	12	0.72	2.137	28	2.3×10^{-4}	6.0×10^{-3}
	R-MMU-211859	biological oxidations	REACTOME	50	0.446	1.873	211	3.1×10^{-4}	7.0×10^{-3}
	GO:0004478	methionine adenosyltransferase activity	Function	3	0.985	1.953	6	2.1×10^{-4}	1.7×10^{-2}

Supplementary Table 2. Gene set enrichment analysis (GSEA) with Gene Ontology (GO) terms and pathways. GC, control mice on Earth; A1G, mice under artificial earth gravity in space; MG, mice under microgravity in space; ‘Pathway ID,’ pathway identifier; ‘GO ID,’ Gene Ontology identifier; ‘GO term,’ Gene Ontology term; ‘Pathway,‘ pathway name; ‘Database,’ pathway source databases; ‘Category,’ the type of term in the Gene Ontology; ‘numGenes,’ the leading-edge genes that drive the enrichment of each category; ‘ES,’ enrichment score, which reflects the degree to which a gene set is overrepresented at the top or bottom of a ranked list of genes; ‘NES,’ normalized enrichment score; ‘Size,’ size of the pathway after removing missing genes in input gene names; ‘*P*-value,’ enrichment *p*-value of each category; ‘*P*adj,’ a Benjamini-Hochberg (BH)-adjusted *p*-value of each category (n = 3 each).

GO term or pathway	Gene Name	Full name	A1G vs. GC		MG vs. A1G		MG vs. GC	
			Fold change	*p*-value	Fold change	*p*-value	Fold change	*p*-value
Oxidative Stress	Mt1	metallothionein 1	0.44	2.8×10^{-1}	0.81	9.9×10^{-4}	1.25	1.5×10^{-1}
	Nqo1	NAD(P)H dehydrogenase, quinone 1	1.61	2.3×10^{-5}	-0.40	7.3×10^{-1}	1.22	2.2×10^{-4}
	Fos	FBJ osteosarcoma oncogene	2.10	1.2×10^{-4}	-0.95	5.6×10^{-1}	1.15	8.0×10^{-3}
	Junb	jun B proto-oncogene	0.36	6.7×10^{-1}	0.56	1.1×10^{-2}	0.93	1.0×10^{-3}
	Ugt1a1	UDP glucuronosyltransferase 1 family, polypeptide A1	0.82	5.0×10^{-5}	0.09	3.5×10^{-1}	0.91	1.0×10^{-7}
	Gsr	glutathione reductase	0.80	3.6×10^{-5}	-0.02	6.3×10^{-1}	0.78	1.3×10^{-4}
	Gstt2	glutathione S-transferase, theta 2	0.92	6.0×10^{-3}	-0.15	8.0×10^{-1}	0.77	2.7×10^{-2}
	Gpx1	glutathione peroxidase 1	0.55	9.0×10^{-3}	0.15	3.5×10^{-1}	0.70	1.1×10^{-4}
	Gclc	glutamate-cysteine ligase, catalytic subunit	1.06	2.8×10^{-5}	-0.38	4.6×10^{-2}	0.68	8.1×10^{-4}
	Txnrd1	thioredoxin reductase 1	0.85	1.0×10^{-3}	-0.27	1.2×10^{-1}	0.58	2.7×10^{-2}
	Hmox1	heme oxygenase 1	0.35	6.4×10^{-1}	0.13	3.8×10^{-1}	0.47	2.0×10^{-1}
	Sp1	trans-acting transcription factor 1	0.32	1.1×10^{-1}	-0.02	8.5×10^{-1}	0.30	6.9×10^{-2}
Biological oxidations	Cyp4a14	cytochrome P450, family 4, subfamily a, polypeptide 14	4.61	2.8×10^{-9}	-0.59	1.0	4.03	3.7×10^{-159}
	Fmo3	flavin containing monooxygenase 3	1.75	2.3×10^{-8}	1.88	6.0×10^{-3}	3.63	1.4×10^{-35}
	Cyp2b9	cytochrome P450, family 2, subfamily b, polypeptide 9	1.99	5.0×10^{-3}	1.43	6.5×10^{-2}	3.42	3.6×10^{-21}
	Cyp2c69	cytochrome P450, family 2, subfamily c, polypeptide 69	0.30	7.3×10^{-1}	2.81	7.7×10^{-4}	3.11	2.9×10^{-10}
	Cyp4a10	cytochrome P450, family 4, subfamily a, polypeptide 10	2.82	1.7×10^{-39}	-0.42	9.1×10^{-1}	2.40	3.2×10^{-6}
	Fmo2	flavin containing monooxygenase 2	0.75	2.6×10^{-2}	1.30	1.0×10^{-3}	2.05	6.7×10^{-16}
	Cyp2c38	cytochrome P450, family 2, subfamily c, polypeptide 38	0.87	6.0×10^{-4}	1.12	2.2×10^{-11}	1.99	1.4×10^{-5}
	Cyp2c40	cytochrome P450, family 2, subfamily c, polypeptide 40	0.18	7.7×10^{-1}	1.77	1.5×10^{-2}	1.94	8.9×10^{-5}
	Sult1e1	sulfotransferase family 1E, member 1	0.05	7.2×10^{-1}	1.74	9.5×10^{-7}	1.78	1.0×10^{-5}
	Cyp26b1	cytochrome P450, family 26, subfamily b, polypeptide 1	2.83	6.0×10^{-5}	-1.05	5.6×10^{-2}	1.77	3.6×10^{-15}
	Cyp2c54	cytochrome P450, family 2, subfamily c, polypeptide 54	1.12	3.0×10^{-5}	0.64	1.9×10^{-5}	1.76	7.9×10^{-32}
	Sult2a7	sulfotransferase family 2A, dehydroepiandrosterone (DHEA)-preferring, member 7	0.61	3.7×10^{-1}	1.08	5.0×10^{-3}	1.69	2.1×10^{-4}
	Cyp2c37	cytochrome P450, family 2. subfamily c, polypeptide 37	0.92	1.2×10^{-4}	0.74	1.5×10^{-7}	1.66	3.4×10^{-28}
	Cyp3a59	cytochrome P450, family 3, subfamily a, polypeptide 59	0.79	2.7×10^{-2}	0.70	2.0×10^{-3}	1.49	1.6×10^{-8}
	Cyp39a1	cytochrome P450, family 39, subfamily a, polypeptide 1	0.83	1.0×10^{-3}	0.63	1.5×10^{-5}	1.46	1.3×10^{-13}
	Cyp2b10	cytochrome P450, family 2, subfamily b, polypeptide 10	1.54	2.2×10^{-7}	-0.11	7.8×10^{-1}	1.43	3.0×10^{-6}
	Cyp2c39	cytochrome P450, family 2, subfamily c, polypeptide 39	0.25	8.1×10^{-1}	1.16	4.2×10^{-5}	1.41	2.7×10^{-5}
	Ces1b	carboxylesterase 1B	0.74	2.0×10^{-2}	0.58	5.5×10^{-6}	1.31	5.1×10^{-9}

Cyp4a31	cytochrome P450, family 4, subfamily a, polypeptide 31	2.69	1.5×10^{-16}	-1.48	5.7×10^{-2}	1.20	4.5×10^{-4}
Cyp2c68	cytochrome P450, family 2, subfamily c, polypeptide 68	0.17	5.2×10^{-1}	0.99	1.8×10^{-12}	1.15	3.9×10^{-11}
Cyp2c29	cytochrome P450, family 2, subfamily c, polypeptide 29	1.32	2.2×10^{-14}	-0.18	2.1×10^{-1}	1.13	1.1×10^{-10}
Mgst3	microsomal glutathione S-transferase 3	1.52	2.4×10^{-5}	-0.44	2.4×10^{-1}	1.08	4.1×10^{-4}
Cyp2a5	cytochrome P450, family 2, subfamily a, polypeptide 5	1.65	5.0×10^{-3}	-0.63	8.0×10^{-3}	1.02	2.9×10^{-7}
Cyp2c50	cytochrome P450, family 2, subfamily c, polypeptide 50	0.84	4.9×10^{-4}	0.15	1.8×10^{-1}	0.99	2.8×10^{-9}
Aldh1a1	aldehyde dehydrogenase family 1, subfamily A1	1.10	4.2×10^{-9}	-0.14	5.4×10^{-1}	0.96	2.0×10^{-7}
Maob	monoamine oxidase B	0.68	4.0×10^{-3}	0.26	2.2×10^{-2}	0.94	5.7×10^{-7}
Ugt1a1	UDP glucuronosyltransferase 1 family, polypeptide A1	0.82	5.0×10^{-5}	0.09	3.5×10^{-1}	0.91	1.0×10^{-7}
Fmo1	flavin containing monooxygenase 1	0.45	9.3×10^{-2}	0.44	4.5×10^{-4}	0.89	2.5×10^{-8}
Gclm	glutamate-cysteine ligase, modifier subunit	0.72	8.0×10^{-3}	0.16	2.3×10^{-1}	0.88	1.2×10^{-4}
Chac1	ChaC, cation transport regulator 1	-0.11	3.7×10^{-1}	0.99	1.0×10^{-3}	0.88	1.7×10^{-2}
Cyp3a13	cytochrome P450, family 3, subfamily a, polypeptide 13	0.48	1.5×10^{-1}	0.38	6.0×10^{-3}	0.86	3.5×10^{-6}
Cyp2e1	cytochrome P450, family 2, subfamily e, polypeptide 1	0.44	2.7×10^{-1}	0.39	8.0×10^{-4}	0.83	3.0×10^{-6}
Nnmt	nicotinamide N-methyltransferase	0.01	1.2×10^{-1}	0.79	1.3×10^{-7}	0.80	1.6×10^{-4}
Cyp3a25	cytochrome P450, family 3, subfamily a, polypeptide 25	0.54	6.7×10^{-2}	0.24	1.1×10^{-1}	0.78	1.9×10^{-4}
Gstt2	glutathione S-transferase, theta 2	0.92	6.0×10^{-3}	-0.15	8.0×10^{-1}	0.77	2.7×10^{-2}
Mat1a	methionine adenosyltransferase I, alpha	0.14	2.6×10^{-1}	0.61	6.2×10^{-8}	0.75	2.3×10^{-5}
Adh4	alcohol dehydrogenase 4 (class II), pi polypeptide	0.47	2.8×10^{-1}	0.26	1.6×10^{-2}	0.72	3.0×10^{-3}
Gclc	glutamate-cysteine ligase, catalytic subunit	1.06	2.8×10^{-5}	-0.38	4.6×10^{-2}	0.68	8.1×10^{-4}
Cyp3a11	cytochrome P450, family 3, subfamily a, polypeptide 11	0.72	5.5×10^{-1}	-0.05	8.3×10^{-1}	0.67	8.3×10^{-4}
Cmbl	carboxymethylenebutenolidase-like (Pseudomonas)	0.49	1.4×10^{-1}	0.18	1.6×10^{-1}	0.67	9.0×10^{-3}
Cyp2d26	cytochrome P450, family 2, subfamily d, polypeptide 26	0.40	3.8×10^{-1}	0.23	3.1×10^{-2}	0.63	5.0×10^{-3}
Cyp17a1	Glutathione S-transferase kappa 1	-0.53	1.5×10^{-4}	1.12	4.3×10^{-2}	0.59	7.2×10^{-1}
Sult1b1	sulfotransferase family 1B, member 1	-0.46	1.7×10^{-2}	1.05	4.1×10^{-6}	0.59	4.2×10^{-2}
Ugt1a6b	UDP glucuronosyltransferase 1 family, polypeptide A6B	0.48	2.1×10^{-1}	0.10	3.3×10^{-1}	0.57	2.5×10^{-2}
Ugdh	UDP-glucose dehydrogenase	0.89	4.0×10^{-3}	-0.34	3.6×10^{-1}	0.55	9.0×10^{-3}
Slc35d1	solute carrier family 35 (UDP-glucuronic acid/UDP-N-acetylgalactosamine dual transporter), member D1	0.89	9.9×10^{-5}	-0.35	1.8×10^{-2}	0.55	5.2×10^{-2}
Sult1a1	sulfotransferase family 1A, phenol-preferring, member 1	-0.03	9.0×10^{-2}	0.57	3.9×10^{-5}	0.54	5.5×10^{-2}
Ces1c	carboxylesterase 1C	0.27	1.0	0.25	2.5×10^{-2}	0.53	4.7×10^{-2}

Rxra	retinoid X receptor alpha	0.30	9.9×10^{-1}	0.19	4.2×10^{-2}	0.49	6.5×10^{-2}
Cyp2j5	cytochrome P450, family 2, subfamily j, polypeptide 5	0.31	7.5×10^{-1}	0.17	1.1×10^{-1}	0.48	6.9×10^{-2}
Ahcy	S-adenosylhomocysteine hydrolase	0.13	1.9×10^{-1}	0.32	6.0×10^{-3}	0.45	1.4×10^{-1}
Slc26a2	solute carrier family 26 (sulfate transporter), member 2	0.51	1.0×10^{-2}	-0.09	7.5×10^{-1}	0.42	2.8×10^{-2}
N6amt1	N-6 adenine-specific DNA methyltransferase 1 (putative)	0.06	5.6×10^{-1}	0.36	1.5×10^{-1}	0.42	4.5×10^{-1}
Cyp51	cytochrome P450, family 51	-0.31	9.4×10^{-4}	0.64	1.6×10^{-5}	0.33	4.2×10^{-1}
Cyp4f14	cytochrome P450, family 4, subfamily f, polypeptide 14	0.17	4.6×10^{-1}	0.17	1.7×10^{-1}	0.33	5.0×10^{-1}
Por	cytochrome p450 oxidoreductase	0.02	3.9×10^{-2}	0.30	1.7×10^{-2}	0.32	7.9×10^{-1}
Nr1h4	nuclear receptor subfamily 1, group H, member 4	0.15	4.3×10^{-1}	0.17	1.4×10^{-1}	0.32	7.0×10^{-1}
Gstz1	glutathione transferase zeta 1 (maleylacetoacetate isomerase)	0.17	2.6×10^{-1}	0.15	1.0×10^{-1}	0.32	8.0×10^{-1}
Cyp3a44	cytochrome P450, family 3, subfamily a, polypeptide 44	0.07	6.5×10^{-1}	0.23	4.8×10^{-2}	0.30	8.1×10^{-2}
Adh6b	alcohol dehydrogenase 6B	-0.06	4.3×10^{-1}	0.33	6.5×10^{-2}	0.27	2.2×10^{-1}
Sult2a8	sulfotransferase family 2A, dehydroepiandrosterone (DHEA)-preferring, member 8	0.08	1.2×10^{-1}	0.17	7.3×10^{-2}	0.25	8.8×10^{-1}
Abhd14b	abhydrolase domain containing 14b	0.02	5.9×10^{-2}	0.23	9.1×10^{-2}	0.25	6.5×10^{-1}
Cyp2d22	cytochrome P450, family 2, subfamily d, polypeptide 22	0.02	5.6×10^{-1}	0.20	2.9×10^{-2}	0.22	1.1×10^{-1}
Mgst2	microsomal glutathione S-transferase 2	-0.39	3.8×10^{-1}	0.60	1.5×10^{-1}	0.22	6.3×10^{-1}
Acsm1	acyl-CoA synthetase medium-chain family member 1	0.07	1.5×10^{-1}	0.14	2.0×10^{-1}	0.21	6.1×10^{-1}
Gstk1	glutathione S-transferase kappa 1	0.01	9.2×10^{-2}	0.17	1.9×10^{-1}	0.19	6.2×10^{-1}
Cyp2d10	cytochrome P450, family 2, subfamily d, polypeptide 10	0.11	3.4×10^{-2}	0.04	1.7×10^{-1}	0.15	2.3×10^{-1}
Mat2a	methionine adenosyltransferase II, alpha	-0.13	1.5×10^{-1}	0.26	1.5×10^{-1}	0.13	9.7×10^{-1}
Marc2	mitochondrial amidoxime reducing component 2	-0.14	5.4×10^{-2}	0.25	7.4×10^{-2}	0.11	4.3×10^{-1}
Cyp2c67	cytochrome P450, family 2, subfamily c, polypeptide 67	-0.16	5.0×10^{-3}	0.25	3.0×10^{-2}	0.09	2.2×10^{-1}
Cyp2b19	cytochrome P450, family 2, subfamily b, polypeptide 19	-0.09	3.9×10^{-1}	0.14	1.9×10^{-1}	0.05	7.9×10^{-1}
Slc35b2	solute carrier family 35, member B2	-0.22	5.5×10^{-2}	0.20	1.9×10^{-1}	-0.02	4.9×10^{-1}

Supplementary Table 3. Differentially expressed genes annotated to Gene Ontology (GO) terms or pathways related to oxidative stress in the livers of control mice on Earth (GC), mice under artificial earth gravity in space (A1G), and mice under microgravity in space (MG). Fold change shows log2 fold change of average gene expression values, and A1G vs. GC indicates the ratio of gene expression in A1G mice to GC mice. Red indicates significantly upregulated genes, whereas blue indicates significantly downregulated genes. Differentially expressed genes were detected by means of DESeq2[8] cloud using a Wald test, a parametric fit type, and setting an adjusted *p*-value cutoff of 0.05 (*n* = 3 each).

GO term	Gene Name	Full name	A1G vs. GC		MG vs. A1G		MG vs. GC	
			**Fold change**	***p*-value**	**Fold change**	***p*-value**	**Fold change**	***p*-value**
Glutathione transferase activity	Gsta2	glutathione S-transferase, alpha 2 (Yc2)	2.43	9.0×10^{-4}	-1.08	5.0×10^{-3}	1.36	9.5×10^{-6}
	Mgst3	microsomal glutathione S-transferase 3	1.52	2.4×10^{-5}	-0.44	2.4×10^{-1}	1.08	4.1×10^{-4}
	Gsta1	glutathione S-transferase, alpha 1 (Ya)	3.73	1.0×10^{-3}	-3.37	6.0×10^{-3}	0.36	5.2×10^{-1}
	Gstm1	glutathione S-transferase, mu 1	1.37	2.1×10^{-1}	-1.07	1.1×10^{-1}	0.30	9.3×10^{-1}
	Gstm2	glutathione S-transferase, mu 2	0.47	2.3×10^{-1}	-0.18	4.0×10^{-1}	0.29	8.8×10^{-1}
	Gstm4	glutathione S-transferase, mu 4	1.21	2.0×10^{-1}	-1.09	2.0×10^{-1}	0.13	4.2×10^{-1}
	Gsta4	glutathione S-transferase, alpha 4	1.03	1.2×10^{-2}	-0.99	6.6×10^{-5}	0.04	2.0×10^{-1}
	Mgst1	microsomal glutathione S-transferase 1	0.04	1.9×10^{-1}	-0.07	4.3×10^{-1}	-0.03	3.7×10^{-2}
	Gstm7	glutathione S-transferase, mu 7	0.44	7.9×10^{-1}	-0.50	7.5×10^{-2}	-0.07	4.8×10^{-2}
	Ltc4s	leukotriene C4 synthase	0.69	3.3×10^{-1}	-1.05	5.1×10^{-2}	-0.36	3.7×10^{-1}
	Gstm5	glutathione S-transferase, mu 5	-0.02	2.5×10^{-1}	-0.40	3.9×10^{-1}	-0.42	5.7×10^{-2}
	Gstm3	glutathione S-transferase, mu 3	1.07	4.6×10^{-1}	-1.63	2.1×10^{-2}	-0.56	8.3×10^{-4}
	Gstm6	glutathione S-transferase, mu 6	-0.03	5.2×10^{-2}	-0.62	3.5×10^{-4}	-0.65	4.5×10^{-7}
	Gstp2	glutathione S-transferase, pi 2	0.87	6.7×10^{-1}	-1.56	2.7×10^{-2}	-0.70	5.4×10^{-2}
	Gstp1	glutathione S-transferase, pi 1	0.18	3.2×10^{-1}	-1.35	4.6×10^{-16}	-1.17	2.4×10^{-20}
Glutathione-mediated detoxification	Gsta2	glutathione S-transferase, alpha 2 (Yc2)	2.43	9.0×10^{-4}	-1.08	5.0×10^{-3}	1.36	9.5×10^{-6}
	Gsta1	glutathione S-transferase, alpha 1 (Ya)	3.73	1.0×10^{-3}	-3.37	6.0×10^{-3}	0.36	5.2×10^{-1}
	Gstm1	glutathione S-transferase, mu 1	1.37	2.1×10^{-1}	-1.07	1.1×10^{-1}	0.30	9.3×10^{-1}
	Gsta4	glutathione S-transferase, alpha 4	1.03	1.2×10^{-2}	-0.99	6.6×10^{-5}	0.04	2.0×10^{-1}
	Anpep	alanyl (membrane) aminopeptidase	-0.06	6.2×10^{-1}	0.04	1.4×10^{-1}	-0.02	3.5×10^{-1}
	Gstm7	glutathione S-transferase, mu 7	0.44	7.9×10^{-1}	-0.50	7.5×10^{-2}	-0.07	4.8×10^{-2}
	Gstm3	glutathione S-transferase, mu 3	1.07	4.6×10^{-1}	-1.63	2.1×10^{-2}	-0.56	8.3×10^{-4}
	Gstm6	glutathione S-transferase, mu 6	-0.03	5.2×10^{-2}	-0.62	3.5×10^{-4}	-0.65	4.5×10^{-7}
	Gstp2	glutathione S-transferase, pi 2	0.87	6.7×10^{-1}	-1.56	2.7×10^{-2}	-0.70	5.4×10^{-2}
	Gstp1	glutathione S-transferase, pi 1	0.18	3.2×10^{-1}	-1.35	4.6×10^{-16}	-1.17	2.4×10^{-20}
Glutathione metabolism	Gsta2	glutathione S-transferase, alpha 2 (Yc2)	2.43	9.0×10^{-4}	-1.08	5.0×10^{-3}	1.36	9.5×10^{-6}
	Mgst3	microsomal glutathione S-transferase 3	1.52	2.4×10^{-5}	-0.44	2.4×10^{-1}	1.08	4.1×10^{-4}
	Gclc	glutamate-cysteine ligase, catalytic subunit	1.06	2.8×10^{-5}	-0.38	4.6×10^{-2}	0.68	8.1×10^{-4}
	Gsta1	glutathione S-transferase, alpha 1 (Ya)	3.73	1.0×10^{-3}	-3.37	6.0×10^{-3}	0.36	5.2×10^{-1}
	Gstm1	glutathione S-transferase, mu 1	1.37	2.1×10^{-1}	-1.07	1.1×10^{-1}	0.30	9.3×10^{-1}
	Odc1	ornithine decarboxylase, structural 1	0.54	1.3×10^{-1}	-0.26	1.3×10^{-1}	0.28	6.7×10^{-1}
	Oplah	5-oxoprolinase (ATP-hydrolysing)	0.44	4.2×10^{-1}	-0.22	2.9×10^{-1}	0.22	9.1×10^{-1}
	Ggt6	gamma-glutamyltransferase 6	0.56	1.4×10^{-1}	-0.41	4.8×10^{-2}	0.16	6.8×10^{-1}
	Gstm4	glutathione S-transferase, mu 4	1.21	2.0×10^{-1}	-1.09	2.0×10^{-1}	0.13	4.2×10^{-1}
	Idh1	isocitrate dehydrogenase 1 (NADP+), soluble	0.36	6.1×10^{-1}	-0.30	2.3×10^{-2}	0.06	9.2×10^{-2}

Gsta4	glutathione S-transferase, alpha 4	1.03	1.2×10^{-2}	-0.99	6.6×10^{-5}	0.04	2.0×10^{-1}
Anpep	alanyl (membrane) aminopeptidase	-0.06	6.2×10^{-1}	0.04	1.4×10^{-1}	-0.02	3.5×10^{-1}
Gstm7	glutathione S-transferase, mu 7	0.44	7.9×10^{-1}	-0.50	7.5×10^{-2}	-0.07	4.8×10^{-2}
Sms	spermine synthase	0.11	7.6×10^{-1}	-0.40	2.9×10^{-1}	-0.29	1.7×10^{-1}
G6pdx	glucose-6-phosphate dehydrogenase X-linked	-0.14	4.1×10^{-2}	-0.18	2.5×10^{-1}	-0.32	7.5×10^{-4}
Gpx6	glutathione peroxidase 6	0.13	7.3×10^{-1}	-0.55	2.0×10^{-1}	-0.42	1.1×10^{-1}
Gstm3	glutathione S-transferase, mu 3	1.07	4.6×10^{-1}	-1.63	2.1×10^{-2}	-0.56	8.3×10^{-4}
Gstm6	glutathione S-transferase, mu 6	-0.03	5.2×10^{-2}	-0.62	3.5×10^{-4}	-0.65	4.5×10^{-7}
Gstp2	glutathione S-transferase, pi 2	0.87	6.7×10^{-1}	-1.56	2.7×10^{-2}	-0.70	5.4×10^{-2}
Nat8	N-acetyltransferase 8 (GCN5-related)	-0.27	3.4×10^{-2}	-0.52	5.7×10^{-2}	-0.78	2.0×10^{-5}
Rrm2	ribonucleotide reductase M2	-0.29	2.9×10^{-2}	-0.56	1.9×10^{-2}	-0.85	3.1×10^{-5}
Pgd	phosphogluconate dehydrogenase	-0.13	1.0×10^{-2}	-0.84	2.6×10^{-6}	-0.96	1.3×10^{-12}
Gstp1	glutathione S-transferase, pi 1	0.18	3.2×10^{-1}	-1.35	4.6×10^{-16}	-1.17	2.4×10^{-20}

Supplementary Table 4. Differentially expressed genes annotated to Gene Ontology (GO) terms or pathways related to oxidative stress in the livers of control mice on Earth (GC), mice under artificial earth gravity in space (A1G), and mice under microgravity in space (MG). Fold change shows log2 fold change of average gene expression values, and A1G vs. GC indicates the ratio of gene expression in A1G mice to GC mice. Red indicates significantly upregulated genes, whereas blue indicates significantly downregulated genes. Differentially expressed genes were detected by means of DESeq2[8] cloud using a Wald test, a parametric fit type, and setting an adjusted *p*-value cutoff of 0.05 (*n* = 3 each).

GO term or pathway	Gene Name	Full name	A1G vs. GC		MG vs. A1G		MG vs. GC	
			**Fold change**	***p*-value**	**Fold change**	***p*-value**	**Fold change**	***p*-value**
methionine adenosyl-transferase activity	Mat1a	methionine adenosyltransferase I, alpha	0.14	2.6×10^{-1}	0.61	6.2×10^{-8}	0.75	2.3×10^{-5}
	Mat2a	methionine adenosyltransferase II, alpha	-0.13	1.5×10^{-1}	0.26	1.5×10^{-1}	0.13	9.7×10^{-1}
Superpathway of methionine degradation	Cbs	methionine adenosyltransferase I, alpha	0.48	2.1×10^{-1}	0.35	7.0×10^{-3}	0.83	9.8×10^{-5}
	Mat1a	cystathionase (cystathionine gamma-lyase)	0.14	2.6×10^{-1}	0.61	6.2×10^{-8}	0.75	2.3×10^{-5}
	Cth	cysteine dioxygenase 1, cytosolic	0.16	4.8×10^{-1}	0.44	2.1×10^{-4}	0.60	1.5×10^{-2}
	Cdo1	sulfite oxidase	-0.03	5.7×10^{-2}	0.63	6.2×10^{-6}	0.60	8.0×10^{-3}
	Suox	S-adenosylhomocysteine hydrolase	0.33	6.6×10^{-1}	0.14	2.3×10^{-1}	0.46	1.2×10^{-1}
	Ahcy	betaine-homocysteine methyltransferase	0.13	1.9×10^{-1}	0.32	6.0×10^{-3}	0.45	1.4×10^{-1}
	Bhmt	propionyl Coenzyme A carboxylase, beta polypeptide	-0.46	9.1×10^{-2}	0.76	2.8×10^{-2}	0.30	9.2×10^{-1}
	Pccb	propionyl-Coenzyme A carboxylase, alpha polypeptide	0.18	3.6×10^{-1}	0.11	2.3×10^{-1}	0.28	8.8×10^{-1}
	Pcca	methionine adenosyltransferase II, beta	0.08	1.8×10^{-1}	0.09	3.6×10^{-1}	0.18	5.6×10^{-1}
	Mat2b	methionine adenosyltransferase II, alpha	0.02	1.4×10^{-1}	0.13	3.5×10^{-1}	0.15	4.7×10^{-1}
	Mat2a	betaine-homocysteine methyltransferase 2	-0.13	1.5×10^{-1}	0.26	1.5×10^{-1}	0.13	9.7×10^{-1}
	Bhmt2	methionine adenosyltransferase I, alpha	-0.10	9.0×10^{-3}	0.13	2.3×10^{-1}	0.03	9.4×10^{-2}
Sulfur amino acid metabolism	Gclm	glutamate-cysteine ligase, modifier subunit	0.72	8.0×10^{-3}	0.16	2.3×10^{-1}	0.88	1.2×10^{-4}
	Cbs	cystathionine beta-synthase	0.48	2.1×10^{-1}	0.35	7.0×10^{-3}	0.83	9.8×10^{-5}
	Mat1a	methionine adenosyltransferase I, alpha	0.14	2.6×10^{-1}	0.61	6.2×10^{-8}	0.75	2.3×10^{-5}
	Cth	cystathionase (cystathionine gamma-lyase)	0.16	4.8×10^{-1}	0.44	2.1×10^{-4}	0.60	1.5×10^{-2}
	Cdo1	cysteine dioxygenase 1, cytosolic	-0.03	5.7×10^{-2}	0.63	6.2×10^{-6}	0.60	8.0×10^{-3}
	Suox	sulfite oxidase	0.33	6.6×10^{-1}	0.14	2.3×10^{-1}	0.46	1.2×10^{-1}
	Ahcy	S-adenosylhomocysteine hydrolase	0.13	1.9×10^{-1}	0.32	6.0×10^{-3}	0.45	1.4×10^{-1}
	Bhmt	betaine-homocysteine methyltransferase	-0.46	9.1×10^{-2}	0.76	2.8×10^{-2}	0.30	9.2×10^{-1}
	Bhmt2	betaine-homocysteine methyltransferase 2	-0.10	9.0×10^{-3}	0.13	2.3×10^{-1}	0.03	9.4×10^{-2}

Supplementary Table 5. Differentially expressed genes annotated to Gene Ontology (GO) terms or pathways related to oxidative stress in the livers of control mice on Earth (GC), mice under artificial earth gravity in space (A1G), and mice under microgravity in space (MG). Fold change shows log2 fold change of average gene expression values, and A1G vs. GC indicates the ratio of gene expression in A1G mice to GC mice. Red indicates significantly upregulated genes, whereas blue indicates significantly downregulated genes. Differentially expressed genes were detected by means of DESeq2[8] cloud using a Wald test, a parametric fit type, and setting an adjusted *p*-value cutoff of 0.05 (*n* = 3 each).

References

1 R Core Team (2021). R: A language and environment for statistical computing. R Foundation for Statistical Computing, Vienna, Austria. https://www.R-project.org.

2 Yu, G., Wang, L.-G., Han, Y. & He, Q.-Y. clusterProfiler: an R Package for Comparing Biological Themes Among Gene Clusters. *OMICS: J. Integrative Biol.* **16,** 284-287 (2012).

3 Young, M. D., Wakefield, M. J., Smyth, G. K. & Oshlack, A. Gene ontology analysis for RNA-seq: accounting for selection bias. *Genome Biol*. **11,** R14; 10.1186/gb-2010-11-2-r14 (2010).

4 Korotkevich, G. *et al. bioRxiv*; 10.1101/060012 (2021).

5 Luo, W. & Brouwer, C. Pathview: an R/Bioconductor package for pathway-based data integration and visualization. *Bioinformatics*. **29,** 1830-1831 (2013).

6 Kanehisa, M. & Goto, S. KEGG: kyoto encyclopedia of genes and genomes. *Nucleic Acids Res.* **1,** 27-30 (2000).

7 Kanehisa, M. Toward understanding the origin and evolution of cellular organisms. *Protein Sci.* **28,** 1947-1951 (2019).

8 Love, M. I., Huber, W. & Anders, S. Moderated estimation of fold change and dispersion for RNA-seq data with DESeq2. *Genome Biol*. **15,** 550; 10.1186/s13059-014-0550-8 (2014).
